# Two Pathways for the Degradation of Orpiment Pigment (As_2_S_3_) Found in Paintings

**DOI:** 10.1021/jacs.2c12271

**Published:** 2023-04-14

**Authors:** Fréderique T. H. Broers, Koen Janssens, Johanna Nelson Weker, Samuel M. Webb, Apurva Mehta, Florian Meirer, Katrien Keune

**Affiliations:** † Conservation & Science Department, Rijksmuseum, Hobbemastraat 22, Amsterdam 1071 ZC, The Netherlands; ‡ Van’t Hoff Institute for Molecular Sciences, University of Amsterdam, Science Park 904, Amsterdam 1090 GD, The Netherlands; § AXIS Antwerp X-ray Imaging and Spectroscopy Laboratory, University of Antwerp, Groenenborgerlaan 171, Antwerp 2020, Belgium; ∥ Inorganic Chemistry & Catalysis, Debye Institute for Nanomaterials Science & Institute for Sustainable and Circular Chemistry, Utrecht University, Universiteitsweg 99, Utrecht 3584 CG, The Netherlands; ⊥ Stanford Synchrotron Radiation Lightsource, SLAC National Accelerator Laboratory, 2575 Sand Hill Road, Menlo Park, California 94025, United States

## Abstract

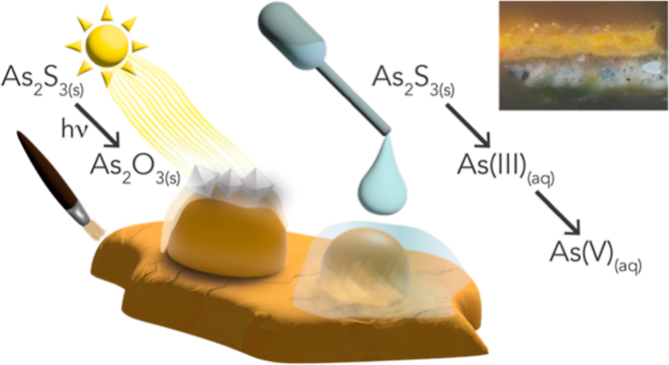

Paintings are complex objects containing many different chemical compounds that can react over time. The degradation of arsenic sulfide pigments causes optical changes in paintings. The main degradation product was thought to be white arsenolite (As_2_O_3_), but previous research also showed the abundant presence of As(V) species. In this study, we investigate the influence of the presence of a medium on the degradation mechanism of orpiment (As_2_S_3_) using synchrotron radiation (SR)-based tomographic transmission X-ray microscopy, SR-based micro-X-ray fluorescence, and X-ray absorption near edge structure spectroscopy. Upon direct illumination of dry orpiment powder using UV–visible light, only the formation of As_2_O_3_ was observed. When As_2_S_3_ was surrounded by a medium and illuminated, As_2_O_3_ was only observed in the area directly exposed to light, while As(V) degradation species were found elsewhere in the medium. Without accelerated artificial light aging, As(V)_(aq)_ species are formed and migrate throughout the medium within weeks after preparation. In both scenarios, the As(V) species form via intermediate As(III)_(aq)_ species and the presence of a medium is necessary. As(V)_(aq)_ species can react with available cations to form insoluble metal arsenates, which induces stress within the paint layers (leading to, e.g., cracks and delamination) or can lead to a visual change of the image of the painting.

## Introduction

Works of art painted with arsenic sulfide pigments are known to be affected by degradation phenomena.^1−7^ These degradation phenomena can manifest themselves in different ways. Optical changes in the paint can be caused by a color loss of the arsenic sulfide pigment and the formation of new chemical species. The degradation can also result in structural changes of the paint such as flaking or chalking. All of these degradation forms can cause aesthetic damage to the works of art.

**Figure 1:**
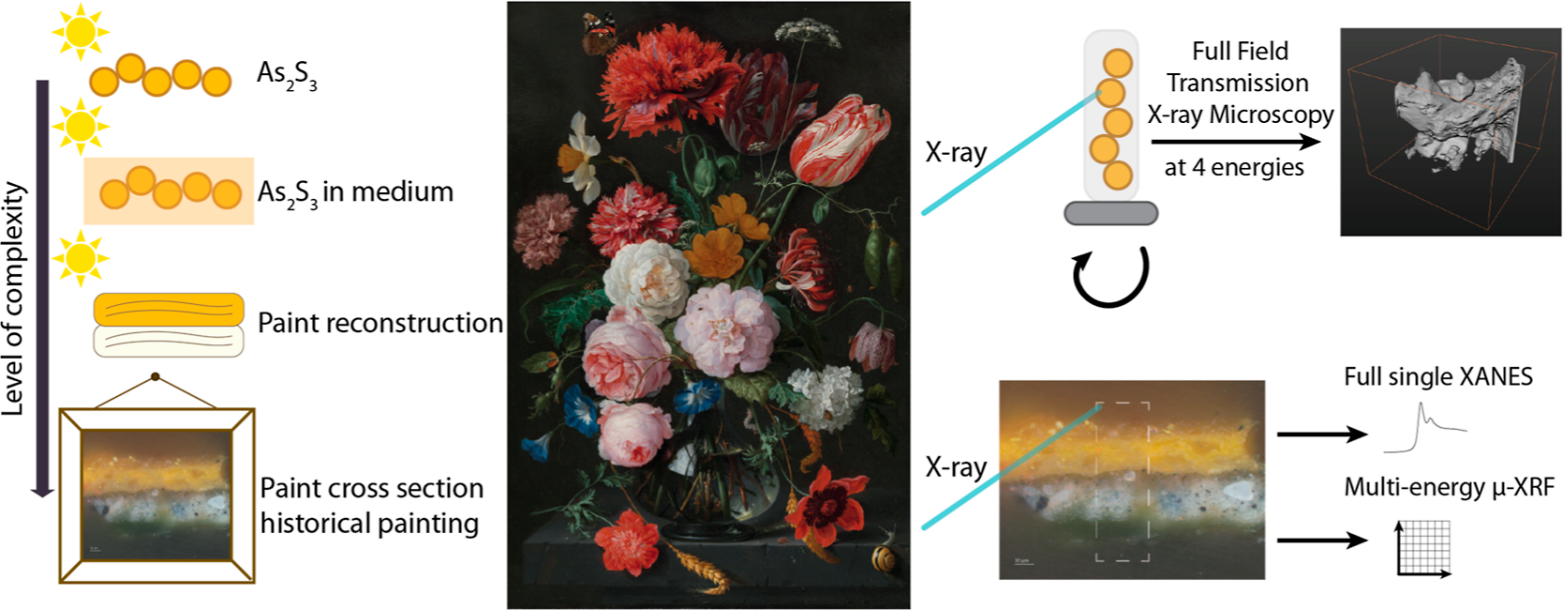
Overview figure of this study. The left part shows the different levels of complexity in samples investigated in this study. The middle shows the oil painting “Still Life with Flowers in a Glass Vase” (1650–1683) by Jan Davidszoon de Heem, Rijksmuseum. On the right, the three main techniques that were used in this study are shown. Measurements were performed at the SSRL. At beamline 6-2c, tomographic full field transmission X-ray microscopy was performed at four energies. At beamline 2-3, full single-point XANES was recorded. Additionally, 2D XRF maps were recorded at 45 energies. These data were used in PCA and clustering to define chemically different regions in the sample based on their XANES fingerprint.

This work focusses on arsenic sulfide degradation in oil paintings. An example of an oil painting affected by the degradation of arsenic sulfide is “Still Life with Flowers in a Glass Vase” (1650–1683) by Jan Davidszoon de Heem (Figure 1, middle). The yellow eglantine rose in the middle of the painting today looks flat and without many features. This is a result of a change in optical effects due to the degradation of the arsenic sulfides that were originally present.^8^ Such changes often result in a loss of depth and of details created by the artist. In this work, the degradation phenomena were studied by investigating the pigment powder, model systems containing the pigment and a relevant paint sample of the painting by De Heem. Figure 1 shows an overview of the work performed in this study.

Artists have been aware of problems associated with arsenic sulfide pigments for a long time. In the medieval text “Mappae Clavicula” (12th century), the incompatibility of arsenic sulfide pigments with some other pigments containing copper and lead was already described.^9^ It is also mentioned in artist manuals such as “De groote waereld in ’t klein geschildert” by Wilhelmus Beurs and “Il Libro Dell’Arte” by Cennino Cennini.^10,11^ However, these archival works only describe reactions that take place on a short time scale and their authors were seemingly not aware of degradation phenomena related to arsenic sulfide observed on longer time scales.

The two main arsenic sulfide pigments are realgar and orpiment. Realgar (As_4_S_4_) is an orange to red pigment, while orpiment (As_2_S_3_) is yellow. Next to these crystalline materials, there exist non-stoichiometric, glassy phases with the general formula As_*x*_S_*y*_.^12−15^ The oxidation state of As is +3 or lower in all these compounds. The earliest documented use of these pigments dates back to ancient Egypt (16th to 11th century B.C.)^16^ and was continued until the end of the 19th century. The high toxicity and their incompatibility with some other pigments were two of the reasons for the decline in popularity of arsenic sulfide pigments.^17^

The degradation of realgar (As_4_S_4_) is described in literature as a photo-induced process taking place upon illumination with visible light; the degradation pathway has been extensively studied. Realgar is thought to undergo a transformation through an intermediate χ-phase (As_4_S_5_), resulting in the formation of a combination of para-realgar (As_4_S_4_) and arsenolite (As_2_O_3_, with As in the +3 oxidation state).^1,12,18−21^ The exact degradation pathway of orpiment has been investigated less intensively. Studies have shown that light exposure results in the direct formation of arsenolite (As_2_O_3_). Vermeulen et al. proposed the breaking of the arsenic–sulfur bond and the release of sulfur as H_2_S in this degradation mechanism.^3^ However, for both realgar as well as orpiment, arsenolite is not the only arsenic-based degradation product. Recent studies on paint cross sections have shown that the presence of arsenic species is not constrained to the paint layer originally containing the arsenic sulfide pigment.^2,4,5^ These X-ray absorption near edge structure (XANES) studies showed that arsenic species with a +5 oxidation state were found in paint layers above and below the original arsenic sulfide paint layer as well as in the varnish layers and, in the case of a painting on a wooden support in the wood structure. These As(V) species derive from the arsenic sulfide pigment and are very mobile. They are therefore able to migrate through all the different paint layers. By using a combination of macro and micro X-ray diffraction (μ-XRD), Simoen et al. have shown that the mobile As(V) species, in the presence of Pb^2+^ ions, can form new, secondary degradation products, such as the lead arsenate minerals mimetite and schultenite.^6,22^ In this study, we took a closer look at the formation of mobile As(V) species in orpiment powder and orpiment containing paints. The main research goal is to address the long-standing question of how the formation of the As(V) species relates to the formation of As_2_O_3_. If we understand under which conditions the As(V) species form, the degradation pathways of orpiment in paintings can be further elucidated. The fate of released sulfur atoms does not fall within the scope of this study. Since all As in arsenic sulfides has a +3 or lower oxidation state, it needs to undergo an oxidation reaction to a +5 state to explain the observation of As(V) species. This oxidation reaction was studied in detail in relation to the removal of toxic arsenic species from groundwater. A study by Lengke et al. describes the rate law of this reaction for the natural orpiment mineral in water of specific pH, measured at 25 °C, as^23^
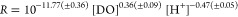
1where *R* describes the rate of orpiment consumption (in mol m^–2^ s^–1^), [DO] is the concentration of dissolved oxygen (in M), and [H^+^] is the proton concentration (M). The proposed activation energy of this reaction is 59.1 kJ/mol at a pH of 7.5 in the temperature range from 25 to 40 °C.^23^ The relatively high value of this activation energy suggests that the oxidation rate of the arsenic sulfide solid is controlled by a surface reaction mechanism and not a diffusion driven mechanism which would require lower activation energies.^24,25^ The presence of As(V) in multiple case studies shows that the reaction conditions in the different objects favor this oxidation reaction. On the basis of preliminary experiments,^26^ we consider that the medium in which the pigment is embedded may play a larger role in the degradation of orpiment than previously anticipated in degradation studies of oil paintings.

To understand the degradation mechanism of orpiment we observe in oil paintings, we took a step back from the (often) centuries-old objects and started by studying the isolated pigment powder. The level of complexity of the model systems was then successively increased (Figure 1, left). For the simple model system, egg yolk was selected as the medium. Egg yolk is used as the binder in the preparation of egg-tempera paint which was used by early Italian renaissance painters such as Giotto and Cimabue.^27^ It was also selected in view of its mainly physical drying process—in contrast to a medium such as linseed oil that features only a chemical drying process. During the chemical drying of linseed oil, more radicals are formed due to higher amount of lipids in linseed oil in contrast to egg yolk. The radicals may influence the degradation of orpiment. For this reason and for the fact that the linseed oil dries much slower compared to egg-tempera,^28^ it was not used as a medium in all but one of the model systems.

With these simple model systems, different relative humidity (RH) conditions were chosen to study the influence of this parameter on the aging process. Some selected models additionally underwent artificially accelerated light aging. Furthermore, experiments with a multi-layered oil paint reconstruction were performed that allowed us to further correlate the observations made in the simple model systems to our observations in works of art. Finally, a micro paint sample from “Still Life with Flowers in a Glass Vase” (Figure 1) from the yellow eglantine rose was examined in order to relate the model system findings to a real case.

Due to the relative low concentration of degradation products in paintings and model samples, these products are often difficult to identify using lab-based analytical techniques. Therefore, a highly focused beam of synchrotron radiation was used for performing spatially resolved measurements at a detection level and (sub)microscopic resolution necessary for studying paint microsamples.^29−34^

XANES analysis was used as the main technique to investigate the different As-species related to the degradation of orpiment in contact with a medium. The X-ray absorption spectrum across the As K-edge is specific for different oxidation states of As, as well as the local coordination of the absorbing atom.^35,36^ We can therefore differentiate between orpiment, As_2_O_3_, and As(V) species using XANES. The XANES data presented in this study were generated at beamline 2-3 at Stanford Synchrotron Radiation Lightsource (SSRL) (CA, USA). XANES measurements were performed (i) on the pure pigment, (ii) on mixtures of orpiment with other pigments, (iii) on the pigment in different media, as well as (iv) on model paint samples and (v) on real paint samples.

Tomographic transmission X-ray microscopy (TXM) at several X-ray energies was used to provide both structural and chemical information on the light-induced aging of orpiment on the nanoscale. TXM was performed at beamline 6-2c at the SSRL (CA, USA), and the data were used to generate a 3D volume rendering of the sample morphology and the distribution of phases within. These data were then correlated to scanning electron microscopy (SEM) images.

By studying the degradation of orpiment under these different conditions, we succeeded in obtaining new information and insights on the requirements for formation of As(V) species and on the competitive reactions giving rise to the formation of As_2_O_3_ and As(V)-species from orpiment.

## Results

**Figure 2:**
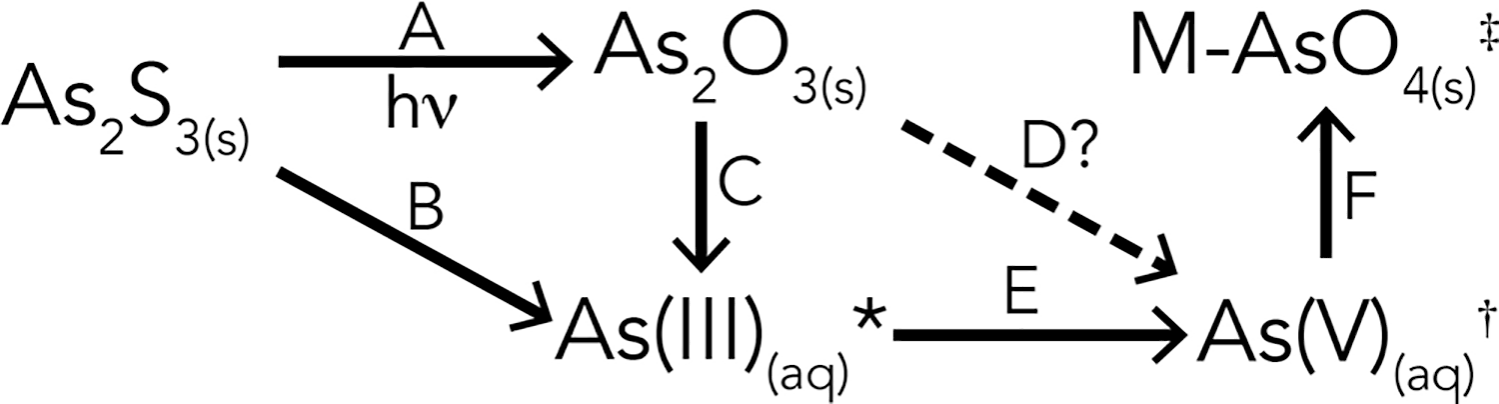
Overview of the different degradation steps possible in orpiment degradation in paintings. In this study, we will argue that reaction step D does not take place. * As(III) (arsenite) species in solution, e.g., AsO_3_^3–^_(aq)_ or a more protonated equivalent such as HAsO_3_^2–^_(aq)_, H_2_AsO_3_^–^_(aq)_, or H_3_AsO_3(aq)_. † As(V) (arsenate) species can have different forms in solution such as H_3_AsO_4(aq)_, H_2_AsO_4_^–^_(aq)_, HAsO_4_^2–^_(aq)_, or AsO_4_^3–^_(aq)_. ‡ Metal arsenates such as PbHAsO_4(s)_ or Pb_5_(AsO_4_)_3_Cl_(s)_.

The different reaction steps that may take place in the degradation of orpiment as described in the literature are summarized in the reaction scheme shown in Figure 2. In this study, we focus on the different reaction steps by using simple model systems consisting of a source of arsenic with or without a medium, and with or without applying artificial light aging. The goal is to establish which of the reactions take place and if so, what local conditions are necessary. Table 1 presents an overview of the different samples and the conditions they were aged in.

**Table 1:** Overview of the Pigment and Model Samples Investigated in This Study^a^

sample name	related reaction step	As-source	medium	relative humidity (%)	light aging
AsSPureLA	A (D)	As_2_S_3_	no medium	0 (N_2_), 11 and 90	yes
AsSPureDark	A	As_2_S_3_	no medium	0 (N_2_), 11 and 90	no
AsSEggLA	A, B, C, D, E	As_2_S_3_	egg tempera	11 and 95	yes
AsSEggDark	B, E	As_2_S_3_	egg tempera	95	no
AsOEggDark	D (C, E)	As_2_O_3_	egg tempera	95	no
AsIIIEggDark	E (C, D)	NaAsO_2_	egg tempera	95	no
AsSPbLA	A, B, C, D, E, F	As_2_S_3_	linseed oil—lead white underground	43	yes

a The sample names are describing the arsenic source, the absence of a medium (pure) or the presence of a medium (egg), and whether the sample was LA or not (dark). Sample AsSPbLA was a mock up paint sample consisting of two oil paint layers on canvas: ground paint layer containing lead white and a layer on top containing orpiment.

### Effects of Light-Aging on As_2_S_3_

To study the effect of light-aging on orpiment (reaction step A), pure orpiment was light aged (LA) using an Opsytec Dr. Gröbel BS-02 irradiation chamber. The orpiment was LA under three different conditions. Two airtight containers were used, maintaining a low (ca. 15%) or high level (around 95%) RH in ambient air. In the third condition, one sample of orpiment was aged under a dry nitrogen atmosphere, that is, at 0% RH. Table 1 gives an overview of the samples and conditions used; more details on the preparation and aging of the samples can be found in the Supporting Information. In addition, to follow the light aging of orpiment with SEM, orpiment on a pin mount was studied before and after light aging (see Figure S1).

**Figure 3:**
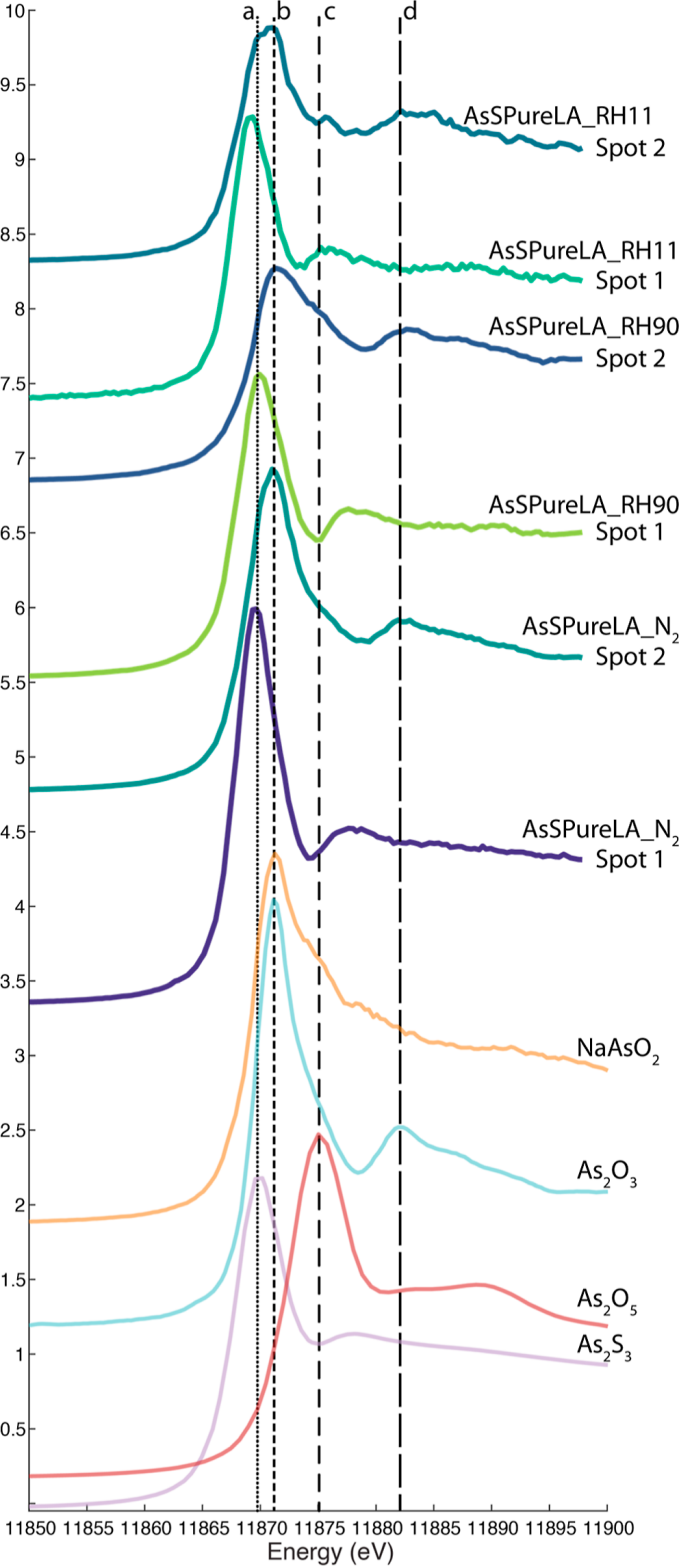
XANES of reference spectra of orpiment (As_2_S_3_), arsenolite (As_2_O_3_), and disodium hydrogen arsenate (Na_2_HAsO_4_), together with XANES measurements taken in sample AsSPureLA_N2, RH11, and RH90. Vertical line “a” indicates the feature specific for As_2_S_3_, line “b” is specific for arsenite species, the combination of lines “b” and “d” is specific for As_2_O_3_ and line “c” for As(V) species.

The samples were studied using single-point XANES, recorded at SSRL beamline 2-3 with a beam size of 2 by 2 μm. These XANES were compared to reference spectra and their locations were chosen to inspect different areas of the sample, that is, in the middle of a pigment particle, at the edge of a particle or further away from a particle. Figure 3 shows the XANES of references spectra for orpiment, arsenolite, and disodium hydrogen arsenate, as well as two locations in the samples AsSPureLA_N_2_, RH11, and RH90. Line “a” indicates the energy where As_2_S_3_ species reach the maximum absorbance of X-rays. Line “b” indicates the same for As(III)–O species, where arsenolite also shows a characteristic post edge feature indicated by line “d”. As(V) species have a slightly higher energy of maximum absorbance indicated by line “c”. Both As_2_S_3_ and As_2_O_3_ were identified in the pure As_2_S_3_ samples, but no indication of the presence of As(V) species was found. The presence of arsenolite was also confirmed with SEM–energy-dispersive X-ray spectroscopy (EDX) (see Figure S2).

The XANES of the light-aged orpiment powder showed no differences for the two different RH levels and the sample aged under nitrogen. In all cases, both areas containing As_2_S_3_ and areas with As_2_O_3_ were observed. We assume that in the sample kept under N_2_, some oxygen was still present within the pigment structure or at the surface of the pigment, leading to the formation of arsenolite upon light aging. Two control reference samples of orpiment, kept in the dark under nitrogen or taken straight from the chemical storage, did not show any formation of arsenolite by XANES, and this was also confirmed by SEM (see Figure S3). We therefore conclude that, in agreement with previous literature, the formation of arsenolite from orpiment is a light-induced degradation process.

**Figure 4:**
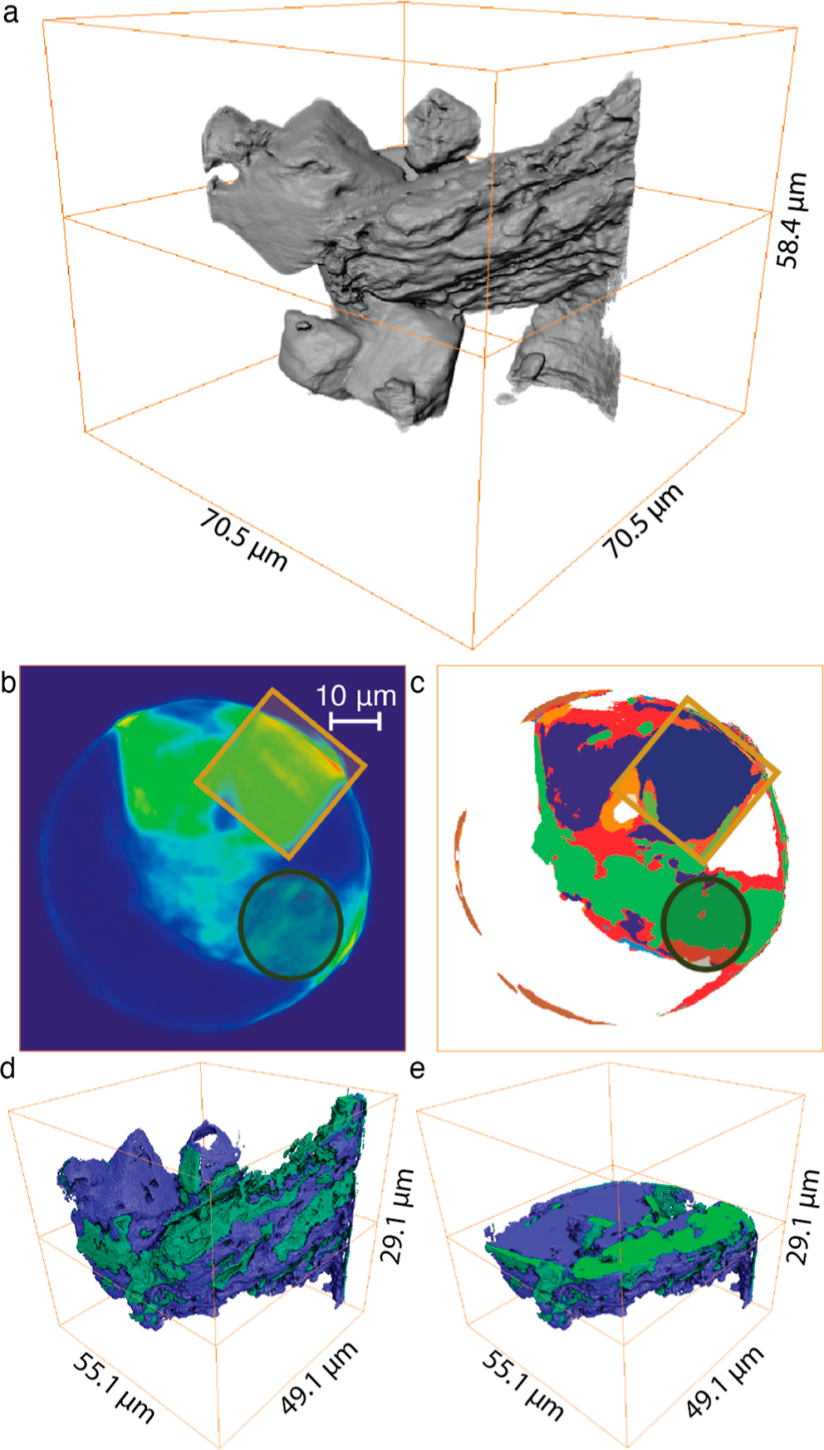
(a) 3D reconstruction of the light-aged orpiment sample imaged during the tomographic TXM experiment performed at an energy of 12,000 eV. (b) Virtual cross section through the tomographic TXM data at 11,875 eV. (c) Results of k-means clustering of the TXM data recorded at four energies: clustering result using six clusters (see the Supporting Information for details) for segmenting the virtual cross section displayed in (b); the square refers to the arsenolite area and the circle to the arsenic sulfide area identified by visual inspection of the 3D sample morphology. (d) 3D rendering of the purple (cluster 5) and green cluster (cluster 3). (e) 3D rendering of the purple and green cluster with an active clipping plane.

The light-aged orpiment (RH 95%) was also studied using tomographic full field TXM at SSRL beamline 6-2c to study the light degradation on the nanoscale. Figure 4a shows the 3D reconstruction of the entire field of view recorded at 12,000 eV, that is, at an energy ca. 100 eV above the As-K edge.

In the middle left and at the top of the reconstruction in Figure 4a, cubic arsenolite crystals can be recognized. These crystals were also observed with SEM, by monitoring the same area of orpiment before and after aging. Images can be found in Figures S1 and S2. The crystals had an octahedral shape, earlier observed by Meirer et al.^37^ The size of the arsenolite crystals observed via TXM varied, in agreement with the SEM images. However, not all of the arsenic sulfide had reacted to form arsenolite crystals. The sample areas not showing arsenolite crystals have a textured surface. From Figure 4a, it cannot be deduced whether this is intact orpiment or orpiment that has undergone some structural changes. A study performed on thermally evaporated thin films of As_2_S_3_ described the alteration of the smooth surface of the thin films as having a “textured appearance” after exposure to radiation from a 380 nm LED source.^38^ The textured appearance in the SEM images of this thin film study is very similar to the textured appearance of the area on the right in Figure 4a. We therefore suspect that this area has also undergone such a structural alteration due to the light aging. This structural alteration was also observed in the SEM study (Figure S1).

Next to these physical changes, we can investigate the chemical changes that took place due to light aging. This was done by repeating the TXM tomography at four energies across the As K absorption edge (11,850, 11,871, 11,875, and 12,000 eV). These values correspond to the baseline ca. 20 eV before the As-K edge (11,850 eV), the white line of As_2_S_3_ (11871 eV), and the characteristic shoulder of As_2_O_3_ (11,875 eV), while at 12,000 eV, well above the As-K edge, all available As is efficiently excited.

Figure 4b shows a virtual slice through the 3D volume at 11,875 eV. Figure S4 shows the virtual slices at all four energies. The four TXM data sets were analyzed using the XANES Wizard software.^39^ Principal component analysis (PCA), followed by a density-based clustering in principal component space using a Gaussian mixture model (GMM) was performed on the same area using all four energies. More details on this analysis can be found in the Supporting Information.^40^

Figure 4c shows the result of the clustering of the data set. The clustering method identified these areas to be different based on the measured absorbance at the four energies. Figure S5b shows the reconstructed four-point “pseudo-XANES” corresponding to the six different clusters. Clusters 2 (blue) and 6 (brown) correspond to areas close to the edge of the particles or to the capillary holder in which the pigment was measured. The reconstructed XANES of the data from these areas are therefore not considered further. Figure 4b shows the transmission image at 11,875 eV; the circle and the square indicate the areas we suspect to be arsenolite (square) and arsenic sulfide (circle) from inspection of the 3D data shown in Figure 4a. The score plot of the two principal components used for the clustering and GMM can be found in Figure S5. The XANES of the purple (cluster 5) and green cluster (cluster 3) contain most data points and also show a chemical difference in their spectral signature. Figure 4d,e shows the 3D reconstruction of the purple and green clusters and Figures S5b and S6a indicate that the ratios of the X-ray absorbance at the energies 11,871 and 11,875 eV are clearly different for these two clusters. Due to the small number of energies recorded, the generated spectra (“pseudo-XANES”) cannot be directly compared to reference XANES. However, the X-ray absorbance values *A*(*E*) measured at the recorded energies can be used to compare the *A*(11,871)/*A*(11,875) ratios with those of reference spectra (Table 2 and Figure S6). This is possible because we are processing voxels (3D pixels) of tomographic data that all have the same thickness, and the absorbance data were normalized to 0 and 1 for each voxel using *A*(11,855) and *A*(12,000 eV), respectively. Table 2 shows the calculated ratios; these data are consistent with our interpretation based on the volume rendering images (Figure S6a).

- The purple cluster mainly identifies the area where octahedral arsenolite crystals are located, characterized by an *A*(11,871)/*A*(11,875) ratio of around 1.3, which matches the value derived from a reference spectrum of arsenolite.
- The green cluster corresponds to the area of the textured orpiment structure and features an *A*(11,871)/*A*(11,875) ratio that is significantly higher (ca. 1.8) and that is in good agreement with the value extracted from an orpiment reference spectrum.

**Table 2:** Normalized X-ray Absorbance at 11,871 and 11,875 eV for the Reference XANES of Orpiment and Arsenolite and the Green and Purple Cluster^a^

	normalized absorbance at 11,871 eV	normalized absorbance at 11,875 eV	*A*(11,871)/*A*(11,875)
XANES of purple cluster	2.183	1.725	1.266
XANES of arsenolite (As_2_O_3_)	1.818	1.429	1.272
XANES of green cluster	1.921	1.051	1.828
XANES of orpiment (As_2_S_3_)	2.178	1.102	1.976

a To compare the experimental and reference data, the ratios have been calculated and shown in the table.

This confirms that the area with the textured appearance consists of orpiment (As_2_S_3_) and the octahedral crystals of the purple clusters are arsenolite (As_2_O_3_) crystals. This analysis allowed us to effectively segment these two phases in 3D at high spatial resolution and using only a limited set of specifically selected X-ray energies, which resulted in significant time savings for the TXM measurement. No significant cluster has a higher absorbance at 11,875 eV than at 11,871 eV, which would be the case if As(V) species were present. This shows that the TXM results are in agreement with the XANES results and do not show the presence of As(V) when pure orpiment powder is light-aged without the presence of a medium. We conclude from the above that the transformation As_2_O_3_ to As(V) needs to take place in the presence of a medium, through reaction steps C and E and not directly via reaction step D.

### As_2_S_3_ Degradation in a Medium, Light-Aged

**Figure 5:**
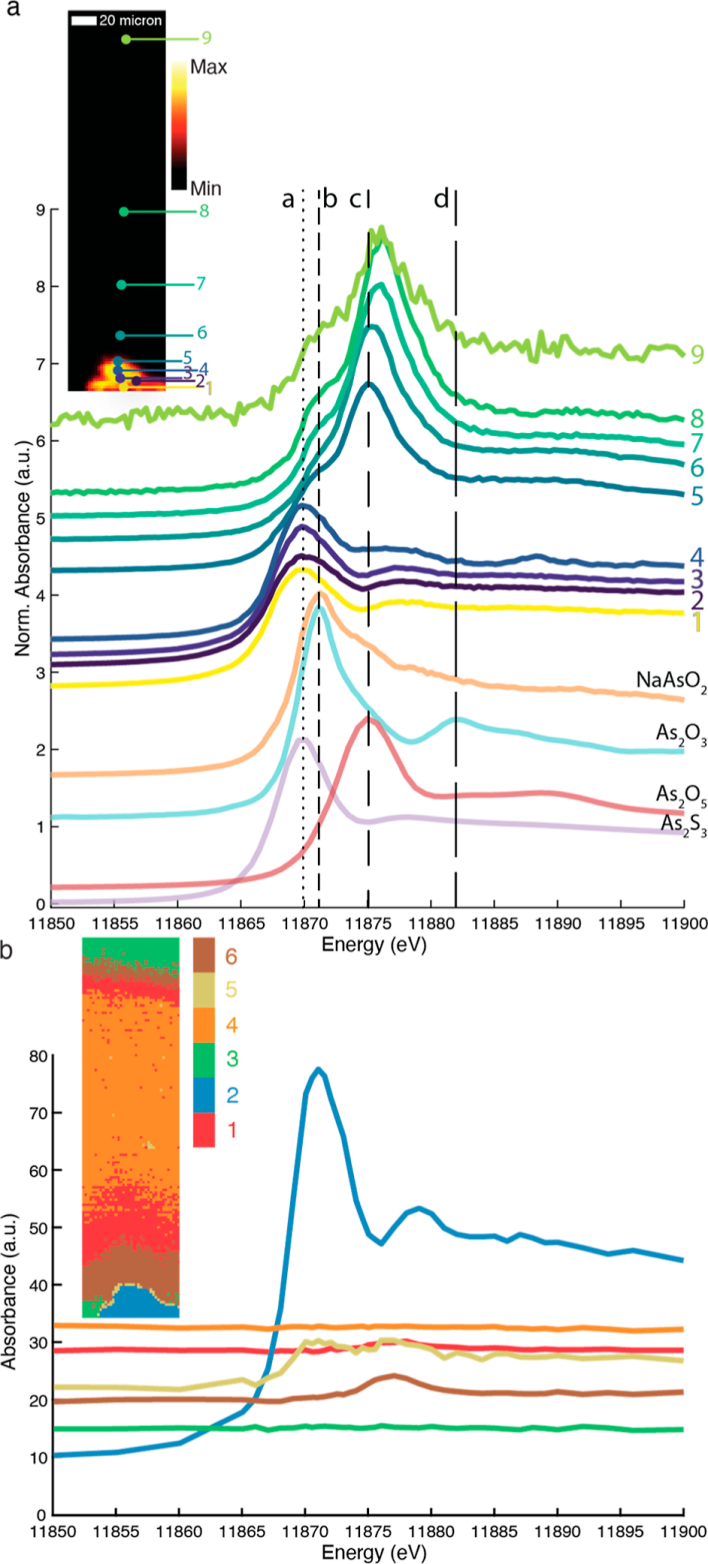
(a) XRF map at *E* = 12,000 eV of a cross section taken from the orpiment in the egg sample kept at low RH; points 1–9 indicate the locations of the point XANES measurements, plotted here in the normalized form. Light aging took place from the top. (b) The inset shows the resulting clustered image of PCA (keeping the first three PCs) followed by k-means clustering using six clusters and subsequent refinement by GMM of the multi-energy XRF maps. The reconstructed XANES of the six clusters are shown in panel (b). The XANES of clusters 1, 2, 5, and 6 show X-ray absorption features above the noise level.

To approximate the (chemical) conditions of a real painting more closely, our next set of model samples consisted of orpiment in a medium. Isolated orpiment grains were divided on a glass slide covered with polytetrafluoroethylene (PTFE) tape. Two to three drops of water–diluted egg yolk were applied on top of the orpiment grains to create a layer of medium in which the degradation products of orpiment (if any) could migrate. The model systems were light-aged for 2 weeks using daylight, UV-A and UV-B lamps from above (see the Supporting Information for more details). The light aging was done at low RH (around 15%) and at high RH (around 95%) in an air atmosphere. After light aging, a sample was taken and embedded in Technovit LC2000 and prepared as cross sections. Thinned down cross sections were installed in front of the beam at beamline 2-3 to acquire XANES and multi energy XRF maps. With this experiment, reaction steps A–E of Figure 2 were studied. The results for the low and high RH samples can be found in Figures 5 and S7, respectively.

Figure 5a shows an XRF map at 12 keV with the measurement locations and the collected, normalized XANES. The XANES measurements show that the arsenic hot spots in the map contained intact orpiment (positions 1–4). Close to the intact orpiment area, the presence of As(V) species was observed (position 5), as indicated by the strong absorbance at 11875 eV (indicated with line “c” in Figure 5a). The XANES clearly show that for both samples containing egg yolk as the medium, As(V) species have formed and migrated into this medium (positions 6–9 in Figure 5a and positions 5–9 in Figure S7a). Measurements taken on the intact orpiment grain (positions 1–4 in Figure 5a) resulted in XANES showing self-absorption effects due to the high local concentration of arsenic.

One of the XANES in the high RH model system (Figure S7b, position 4, in light brown) showed a shift to slightly higher energy that could indicate the presence of arsenolite. Since this spectrum was collected at the top edge of the original pigment grain, this area was directly exposed to light aging. This could explain the light-induced formation of arsenolite at this location.

μ-XRF maps were recorded at 45 energies across the As K-edge. These maps were analyzed using PCA, followed by k-means clustering, subsequently refined by GMM using expectation maximization (EM). The non-normalized XANES, reconstructed from the clustering result, show the presence of intact orpiment (blue cluster). At the interface of the orpiment and medium (yellow cluster), there is a combination of As(III) species and As(V) species. Further into the medium, in the brown and red cluster, we find As(V) species. Comparing Figures 5b and S7b, there does not seem to be a significant difference in the migration pathlength of the As(V) species from the original pigment between the samples aged at low and high RH. If the migration was moisture/RH-driven, we would expect a clear difference in the distance of the As(V) species to the original arsenic source. The averaged XANES of the clusters in either of the samples did not show the presence of any arsenolite.

### As_2_S_3_ Degradation in a Medium in Darkness

A similar experiment was done without exposure to light. This was done to ascertain whether or not the oxidation from As_2_S_3_ to As(V) takes place without exposure to light (reaction steps B and E). The model system was kept in a box at high RH (90%). The box was kept in a dark drawer for 14 days before a sample was taken, embedded, and again shielded from light. The sample was again studied by means of a combination of single point XANES and XRF maps collection at different energies.

**Figure 6:**
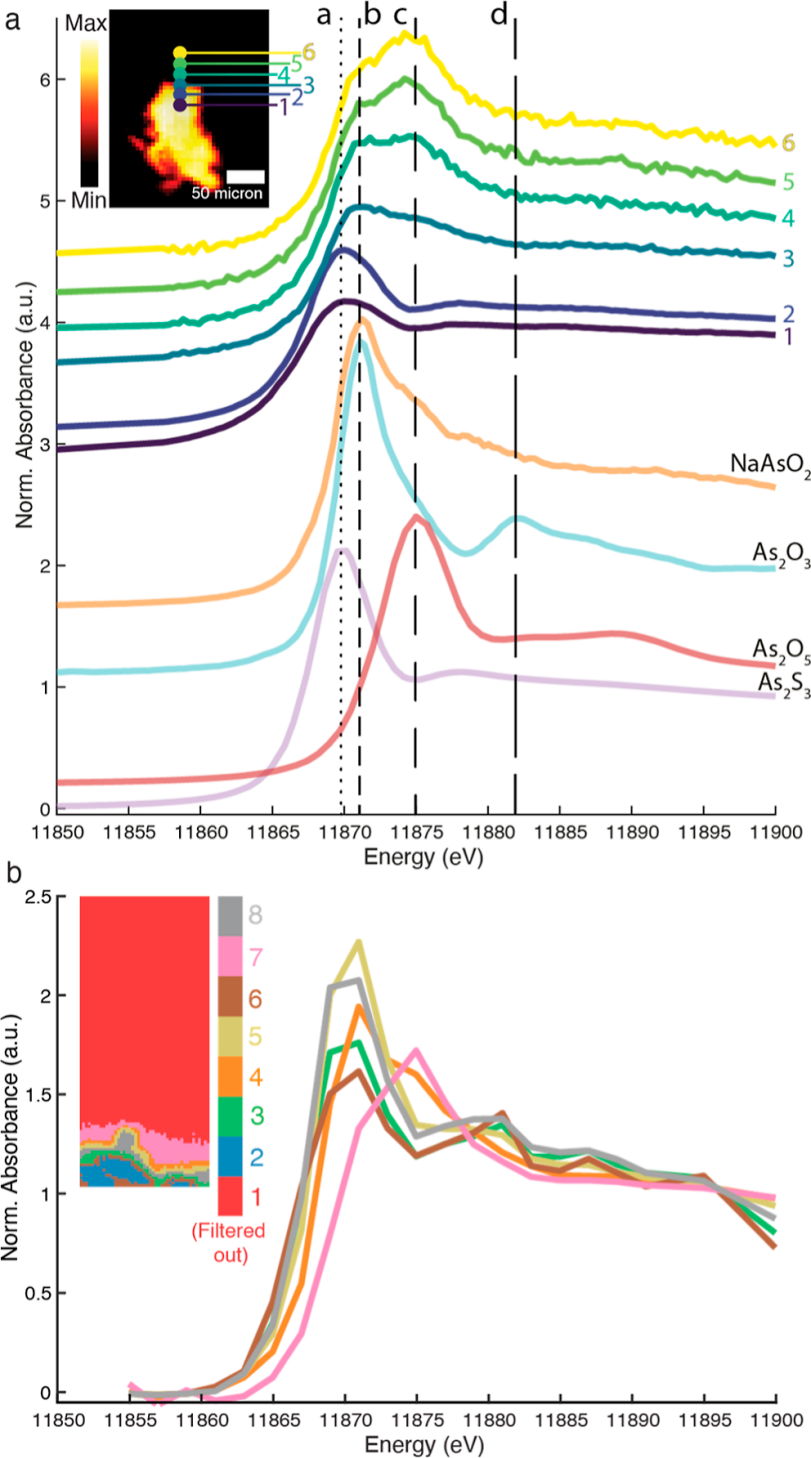
(a) Inset shows the XRF map at 12,000 eV of As_2_S_3_ grains in a medium of egg tempera. The positions where point spectra have been recorded are indicated. The normalized XANES confirm the presence of both As(III) and As(V) species. (b) Clustered image based on PCA and k-means/EM–GMM clustering. The first 4 PCs and 8 clusters were used. The graph shows the reconstructed and normalized XANES of clusters 3–8. Pixels belonging to cluster 1 are now shown as they showed a negligible As-signal. Normalization of the XANES of the blue cluster (2) failed due to strong self-absorption in that region and was removed for clarity. The non-normalized sum XANES for each cluster can be found in Figure S8.

The results of Figure 6 show that also in darkness, As(V) species have formed and migrated into the medium around the pigment grains. Figure 6a shows XANES measurements taken at six locations from the orpiment into the medium. The XANES taken in positions 3–6 show the shift of the white line to a slightly higher energy, related to an arsenic–sulfur species converted into to an arsenic–oxide species (As(III)_(aq)_).^35^ Positions 4–6 also show the formation of As(V) species in the medium. The XANES in positions 3–6 do not show the formation of crystalline arsenolite species, which can be recognized by the characteristic post edge feature indicated by line “d” in Figure 6a.

The results of PCA and k-means/EM–GMM clustering are shown in Figure 6b. The reconstructed XANES of the orange cluster 4, at the interface of the arsenic sulfide grain and the medium shows a slight white line shift to a higher energy. This is consistent with a transition phase from orpiment (As_2_S_3_) toward more oxidized species, probably in the form of an As(III)-oxide. The pink cluster 7 shows the presence of As(V) species. These results show that reaction steps B and E of Figure 2 take place in darkness within a few weeks.

### As_2_O_3_ in a Medium

**Figure 7:**
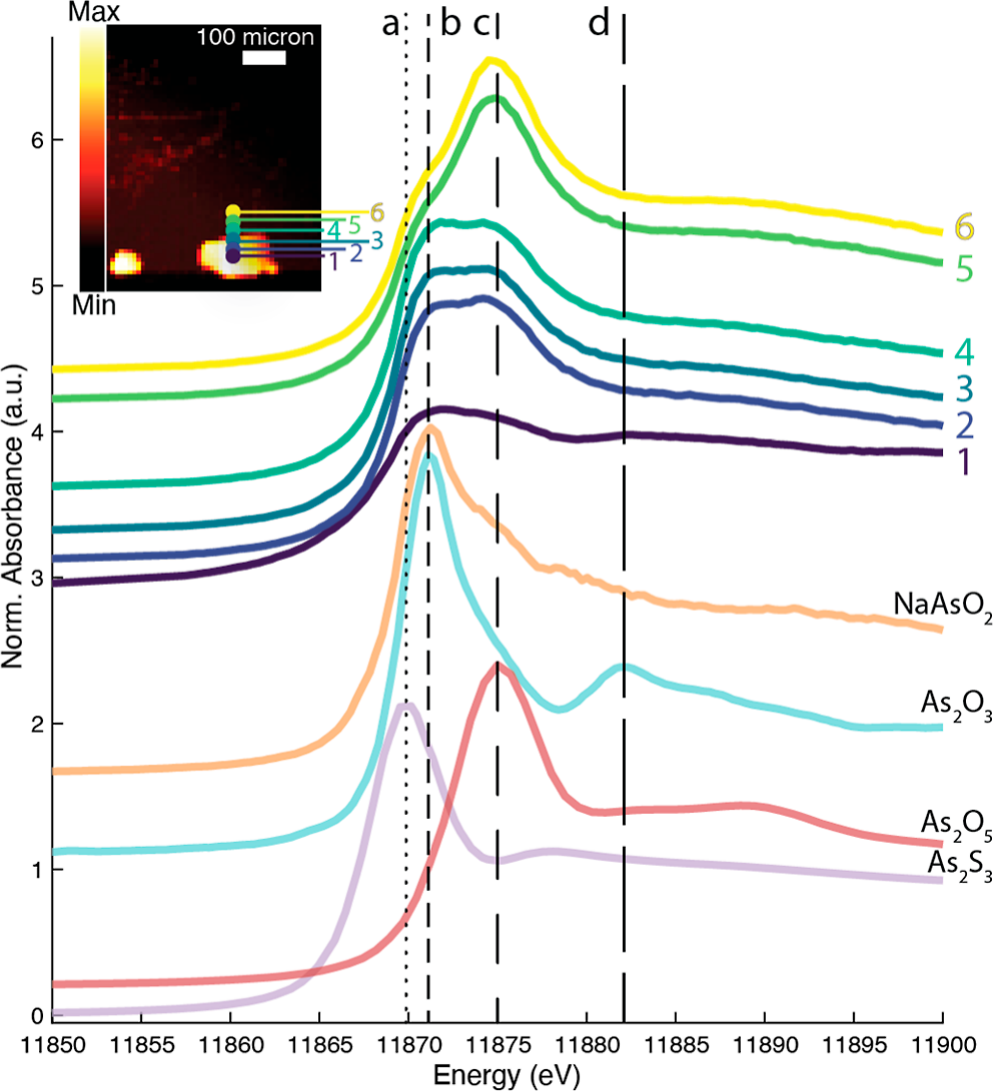
Inset shows the XRF map at 12,000 eV of two As_2_O_3_ grains in an egg tempera medium. Points 1–6 indicate the locations where full X-ray absorption spectra were recorded. Normalized XANES are shown indicating the presence of As(III) and As(V) species.

To study reaction steps C, D, and E (Figure 2), a model sample of arsenolite in egg tempera was used. The inset in Figure 7 again shows the XRF map of a single arsenolite grain at 12,000 eV. In comparison to the equivalent XRF map for the orpiment in egg sample, the local As concentration is higher. Figure 7 shows the XANES recorded at positions 1–6 indicated in the inset. XANES number 1 was recorded in an As hotspot where we expect arsenolite to be located. Self-absorption is again visible in this highly concentration location therefore the XANES has a lower white line absorbance than expected. The XANES recorded in positions 2–4 show a combination of As(III) and As(V) species. In addition, outside of the As hotspot, the XANES data show that As(V) species were present. The relatively high concentration of As in the medium and the XANES of positions 2–4 suggest that some of the arsenolite had dissolved in the water-diluted egg yolk (since orpiment is less soluble in water than arsenolite, this phenomenon was not observed in the previous experiment^41,42^).

Literature shows that during solution of arsenolite, first hydrated arsenite species form, after which oxidation to arsenate species takes place.^41^ The same seems to take place in the model systems with egg tempera. This would suggest that the first reaction step C takes place (As_2_O_3_ > As(III)–OH), followed by reaction step E [As(III)–OH > As(V)] (see Figure 2). The data suggest that As(III) components are present, likely one or more As(III)–OH species. However, since such species cannot be obtained in the isolated form, it was not possible to record a XANES reference spectrum from them to be included in a reliable quantitative fitting model and we remain with a qualitative analysis of the XANES fingerprints.

### Degradation of As(III) Species in a Medium

To study reaction step E (Figure 2), an As(III) salt [sodium(meta)arsenite, NaAsO_2_] was used as the As-source. Similar to the model samples discussed above, an egg tempera medium was used to cover the arsenite salt. The results of the XRF map at 12,000 eV and the recorded XANES spectra can be found in Figure S9. The XRF map shows that the local concentration of arsenic is rather high in the egg tempera and that its distribution is rather homogeneous, consistent with the high solubility of this Na-salt. No remaining As-containing particles could be distinguished as was the case for the samples containing orpiment or arsenolite. The XANES show that both As(III) are still present while new As(V) species have formed. This proves again that the reaction step E takes place within this model system setup without the necessity of exposure to light.

### As(III)-Based Degradation in Other Media

Sodium(meta)arsenite was also aged in another type of medium, dammar (natural resin). The results are shown in Figure S10 and show again the formation of As(V) species and migration in the medium within weeks. Additionally, orpiment in Paraloid-72 (acrylic resin) was LA and a similar trend was observed as found in the sample with egg tempera, namely, the formation of As(III)–OH, followed by the formation and migration of As(V) species. The results are shown in Figures S11 and S12.

### As_2_S_3_ Degradation in a Paint Reconstruction

**Figure 8:**
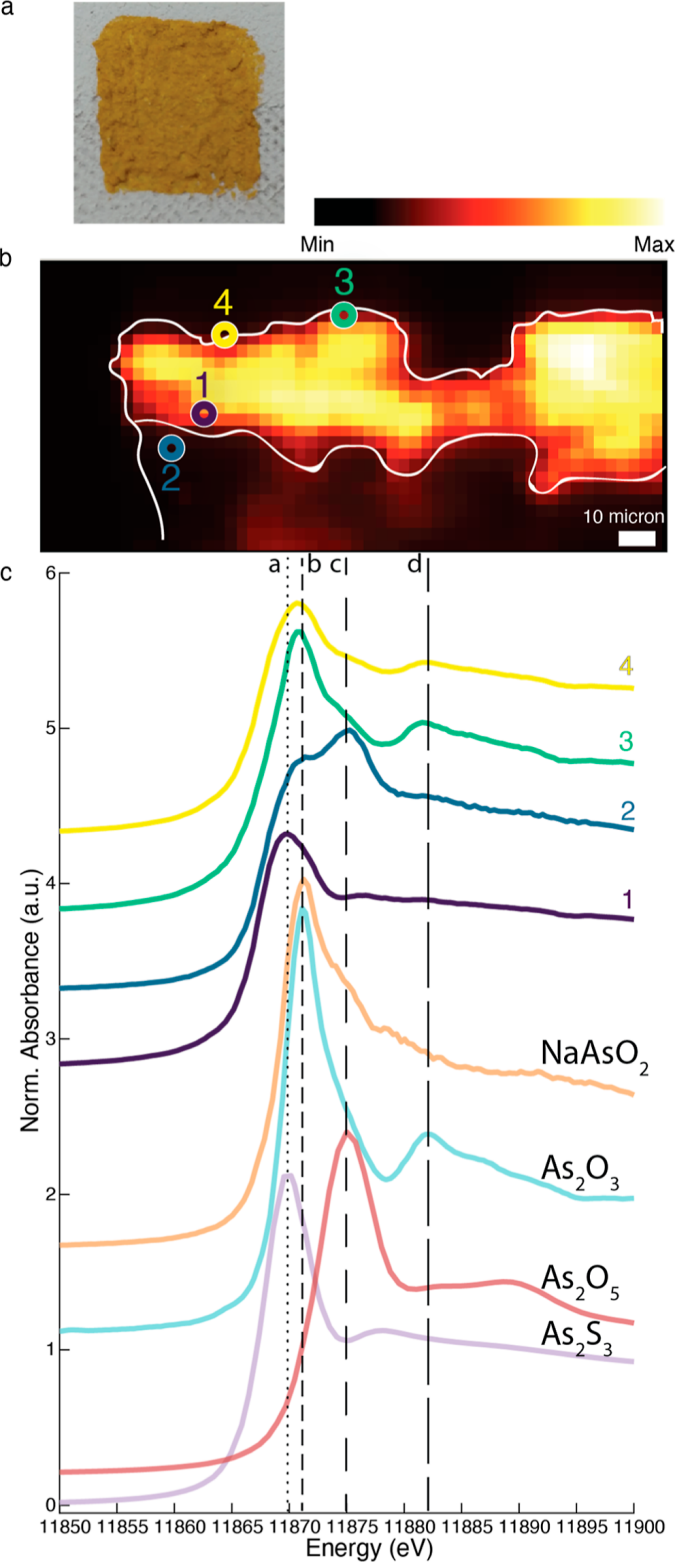
(a) Results obtained from mock-up studies of an orpiment paint layer on a lead white ground layer. (b) XRF map at 12 keV of a cross section of one of the paint reconstructions shown in (a). Points 1–4 indicate the locations where full X-ray near edge absorption spectra have been recorded. (c) The corresponding normalized XANES 1–4. Spectrum 2 was taken in the ground layer consisting of lead white.

To approximate as closely as possible the situation as encountered in oil paintings, an oil paint mockup sample consisting of an orpiment paint layer on a lead white ground layer was studied (Figure 8a). The technical procedure is described in the Supporting Information. Cross sections of the paint reconstructions were taken for analysis. Light microscopy clearly showed the formation of an altered layer on top of the paint reconstruction, exposed to the light. Attenuated total reflection infrared spectroscopy (ATR-IR) (Figure S13) shows the presence of As–O bonds in the top region of the reconstructions, indicating that As_2_O_3_ had formed here. On the other hand, in the bottom part of the orpiment paint layer and in the ground layers, no As–O bonds were detected. Macro X-ray powder diffraction (MA-XRPD) and synchrotron-based μ-XRD also confirmed the presence of arsenolite in the paint reconstructions, but no lead arsenates were detected (see Figure S14, μ-XRD data not shown).

XANES measurements performed on a cross section of the paint reconstruction confirmed that As_2_O_3_ formed at the top of the light-exposed orpiment paint layer, as can be recognized by the combination of lines “b” and “d”. Figure 8b shows the μ-XRF map taken at 12,000 eV with the locations of the XANES measurements indicated.

Four XANES were measured in different positions of the cross section and are shown in Figure 8c. Spectra 3 and 4 were taken at the top of the paint layer and show the typical XANES of arsenolite. Deeper in the orpiment paint layer, intact arsenic sulfide was found (position 1). In the ground layer of the reconstruction (position 2), clearly As(V) species were found. Once again, arsenolite only formed at/near and in the surface of the paint reconstructions, that is, under direct exposure to light. As(V) species formed in and/or migrated toward the lower parts of the paint reconstruction that were not directly exposed to light. This shows that reactions steps A–E take place in this sample. We suspect the absence of lead arsenates (reaction step F) can be either due to the young age (2 years old) of the samples in comparison to the oil paintings in which lead arsenates were found (more than 300 years old),^6,22^ or the concentration of formed lead arsenates is below the detection limit of XRD. The lead phase composition could also have an influence on the kinetics of the reaction, as different lead phases will have different solubility and reactivity in oil paintings. Although in both the reconstruction and the historic painting lead white was present, the composition of this pigment can vary.

### As_2_S_3_ Degradation in a Historical Paint Sample

**Figure 9:**
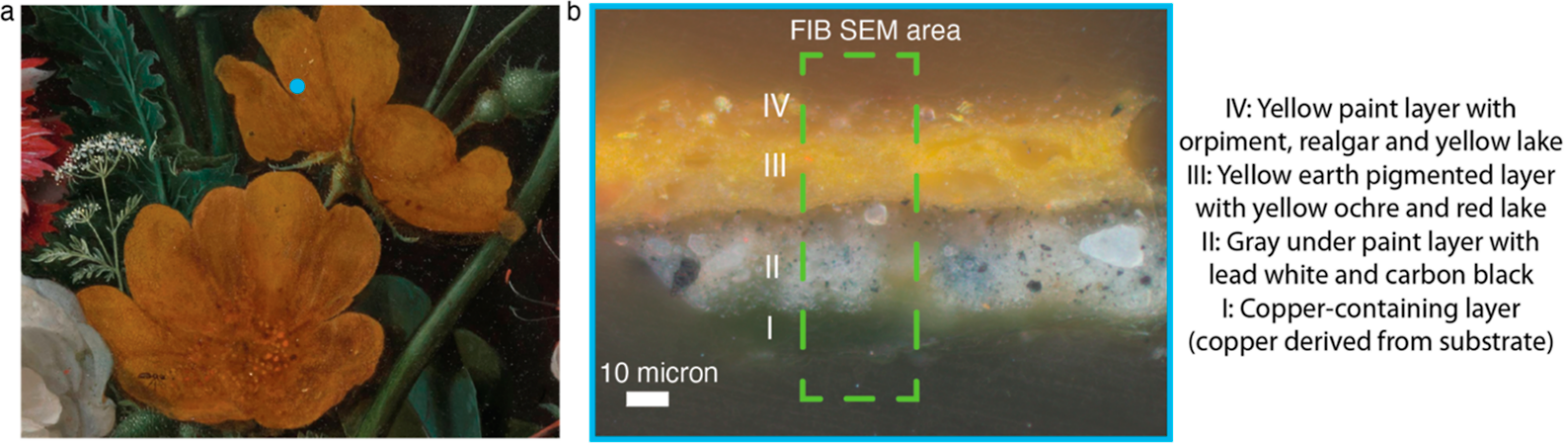
(a) Detail of the yellow eglantine rose in “Still Life with Flowers in a Glass Vase” (ca. 1650–1683) by Jan Davidsz. De Heem, Rijksmuseum (SK-C-214). The blue dot shows the location where a paint cross section was taken in 2001. (b) Dark field light microscopy image taken of the cross section from the yellow eglantine rose.

The Dutch painter Jan Davidszoon de Heem (1606–1684) often used orpiment together with realgar in many of his still life paintings. In the “Still Life with Flowers in a Glass Vase” (ca. 1650–1683, oil on copper plate), in the collection of the Rijksmuseum, Amsterdam, he used arsenic sulfide pigments in the yellow eglantine rose that is shown in Figure 9a. The yellow flower now appears very flat (that is, without any three-dimensional shape) and without the expected anatomical details in the center of the flower. A paint cross section was taken from this yellow flower to study the buildup of the paint layers and for further analysis of the As-containing paint. Figure 9b shows a light microscopy image of the paint sample.

**Figure 10:**
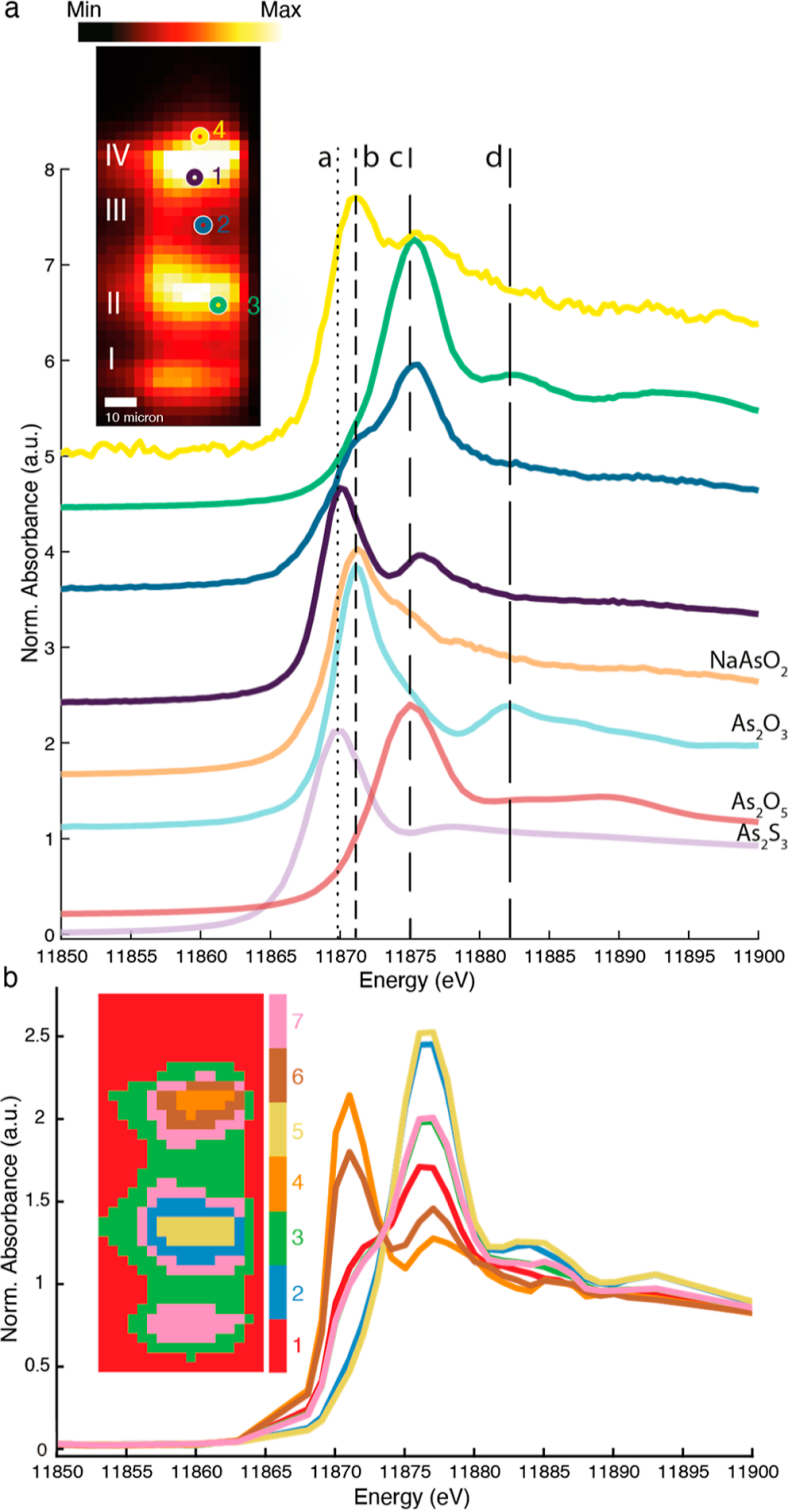
(a) XRF map at 12 keV of a cross section taken from the yellow eglantine rose. 1–4 indicate the locations where XANES plotted in panel a have been recorded. (b) Reconstructed and normalized XANES of the seven clusters resulting from PCA and clustering of the hyperspectral image data. The clustered image is displayed in the inset.

The paint sample consists of four layers. The lowest layer (I) corresponds to the copper substrate, while layer II is a gray underpainting containing the pigments lead white and carbon black. The third layer is a yellow earth pigmented layer with yellow ochre and red lake. The top layer, layer IV, is a yellow paint layer containing orpiment and yellow lake and possibly realgar. The dimension of this paint cross section was decreased by FIB–SEM (see the Supporting Information) and mounted on a pin. The cross section was then studied with XANES and μ-XRF at SSRL beamline 2–3. Several single-point XANES were recorded for this sample. As before, the inset of Figure 10a shows the XRF map at 12,000 eV and the locations of spectra 1–4. Spectrum 1, taken in the arsenic sulfide pigment layer, shows the presence of not only some intact orpiment but also a weaker white line signature of As(V) species. Spectrum 2 shows a combination of As(III) and As(V) species in which the As(V) contribution is more important than in spectrum 1. Spectrum 3, recorded in layer III shows the presence of only As(V) species. We suspect that secondary degradation products in the form of a lead arsenates may have formed here.

Lead arsenates can be formed due to the reaction between hydrated arsenate ions and available lead ions originating from lead white. Due to a lack of reference XANES of different lead arsenates in the pure form, we were not able to obtain more information on the exact species via linear combination fitting of XANES spectrum 3. However, since the minerals schultenite [PbHAsO_4_] and mimetite [Pb_5_(AsO_4_)_3_Cl] have been identified as secondary degradation products in several 17th century in oil paintings, we can assume that also here these species will be present.^6,8,22^ Spectrum 4 was taken at the top of the orpiment paint layer, at the surface of the painting, causing it to be noisier than the other spectra from this sample. The XANES again shows the contribution of As(III) and As(V) species. The former shows a slight shift in location of the white line toward higher energy, which could indicate the presence of arsenolite. In view of the lower signal-to-noise ratio, it was not possible to perform a reliable fitting on this spectrum to verify the agreement with an arsenolite reference spectrum.

45 μ-XRF maps were taken across the arsenic K-edge, enabling to further examine the cross section. PCA and k-means clustering resulted in seven clusters (Figure 10b). The clustering did not identify an arsenolite layer at the top of the cross section. The orange and brown clusters of pixels (layer VI) show the most intense As(III) signals with some weaker contributions from As(V) species. The XANES signature of As(V) species is also present throughout the entire paint stack. The normalized XANES of clusters 2 (blue) and 5 (yellow) are clearly different from those of clusters 3 (green) and 7 (pink). The blue and yellow cluster, corresponding to the lead-containing paint layer III, show the highest As(V) contribution. We expect that the aforementioned lead arsenates mimetite and/or schultenite formed here.

## Discussion

As(V) species were found in all (model) samples that consisted of an arsenic (III) source (orpiment, arsenolite, or an arsenite salt) in contact with a medium (egg tempera). Part of the experiments were also performed with a different type of medium (dammar, natural resin and Paraloid-72, and acrylic resin) and the same trend was observed (Figures S10–S12). In the case where pure (dry, no medium) orpiment powder underwent light-aging, no As(V) species were formed. On the other hand, the formation of arsenolite (As_2_O_3_) is less evident when we performed artificial light aging in the presence of a medium. If arsenolite formed in a model system that had been subjected to artificial light aging, the arsenolite only formed in the regions that had been exposed to light directly. These observations suggest two different pathways for the degradation of orpiment.

- If the orpiment is in a “dry” environment and exposed to light, the formation of arsenolite crystals was observed. The arsenolite crystals formed within 2 weeks of exposure to UV-B light. Previous research using monochromatic light suggest that arsenolite can form within minutes of exposure.^20^ The areas of light-exposed “dry” orpiment which do not show the presence of arsenolite, do appear to have undergone a structural change in the surface of the material. This textured surface of LA arsenic sulfide was previously observed in As_2_S_3_ thin films. X-ray absorption spectroscopy of these structured areas suggests the presence of As_*x*_S_*y*_ species. XANES that were recorded for the light-aged orpiment suggest that As_2_S_3_ was still present in the sample. However, at this stage we cannot unambiguously distinguish whether these spectra originate from a textured surface or an area that was shielded from light, that is, not affected by light-aging. In any case, no As-oxidation was observed in these “dry” conditions.
- If the arsenic species were in contact with a medium. XANES measurements of As_2_S_3_ that was brought into contact with egg tempera in the dark confirmed the formation of As(V) species within 2 weeks, indicating an oxidation. No arsenolite was detected in this sample. The As(V) species were shown to be very mobile in the medium, consistent with previous research that reported As(V) species to be present throughout the multilayer system of oil paintings in which orpiment was used.^4,5^

The next step was to study the behavior of orpiment in a paint system. A model system and a case study containing orpiment that were exposed to artificial and natural light showed that in these situations both arsenolite species and As(V) species form. The orpiment in paint directly exposed to light showed the formation of arsenolite. If we assume that also no oxidation of the original sulfide ions takes place, the reaction taking place could be
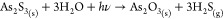
2

However, if on the other hand, as recently suggested by Mirazimi et al.,^42^ there is an oxidation of sulfur to thiosulfate species taking place (i.e., a partial sulfur oxidation), we obtain as overall reaction
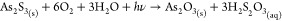
3In case the orpiment containing paint was not exposed to light, no arsenolite was found, while the binding medium area surrounding the orpiment showed the presence of As(V) species. In the previous research, the hypothesis was that the As(V) species evolved from the As_2_O_3_ species.^4^ However, in most of our samples, we found intact orpiment and As(V) species in close proximity to each other, without any As_2_O_3_ present.

Recently, leaching tests have been performed to investigate the arsenic and sulfur species released from orpiment in aqueous solutions.^42,43^ Arsenites (AsO_3_^3–^ or polymers of this ion) were identified as the most abundant As species, while no arsenolite was identified.

Based on these new findings and our own experimental results, we hypothesize that some of the orpiment dissolves as a result of the interaction between As^3+^-ions (having a Lewis acid character) in the orpiment and Lewis bases available in the medium surrounding the grains (such as the OH-groups of lipid molecules), giving rise to arsenite species that remain in solution (and thus do not form arsenolite). These arsenite ions can then diffuse into the binding medium where they may be further oxidized to the arsenate species. When the latter encounter suitable metal counter-ions, the precipitation of several metal-arsenates can take place. In case of the formation of As(V) species, we suggest that the following overall reaction takes place
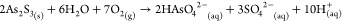
4

[In this equation, the HAsO_4_^2–^ can also be other (protonated) forms of an arsenate (ion) such as AsO_4_^3–^, H_2_AsO_4_^–^, or H_3_AsO_4_.] At the interface of the orpiment and the paint medium, a conversion to As(III)–O species is observed, while most XANES show a combination of As(III) and As(V) species to be present.

We suspect that the interface between the orpiment and the paint medium contains mainly arsenite species. Due to the design of the paint mockup samples and the beam size, we probe a volume of the sample that contains several As-species. Especially in this interface region, the step size of 2 μm is too large to observe all intermediate species as spatially separated. In addition, deconvolution of the XANES in this area is difficult due to a transition from a very highly As-concentrated area (pigment grain) to a low As-concentrated area (paint medium).

In our aging experiments the RH was set at either ∼15 or ∼95%. No clear differences were observed between these conditions on the time scale of our experiments. The presence of some moisture seems to suffice to allow the As(III) > As(V) oxidation reaction to take place. To further investigate the limits of the level of humidity for the oxidation to take place, future research is necessary. As it is improbable to encounter such very low or very high levels of humidity in a painting’s environment, this was not within the scope of our research.

As(V) species can form metal arsenates with different cations, among which Pb^2+^, Fe^2+^, and Ca^2+^. All of these cations are very commonly encountered in historical oil paintings. Painters of different historical periods were already aware of the dangers of mixing arsenic sulfide pigments with pigments containing the above-mentioned cations.^9−11,44^ However, it appears that even when As-based pigments are used in different paint layers, the migration of As(V) species leads to the formation of these new metal arsenate species in different areas of the paint system. The formation of secondary (crystalline) degradation products can cause different problems in a painting. If the formation takes place between paint layers, mechanical stress will be induced in this area, increasing the risk of crack formation or delamination. In the case where the new species form close to the surface of the painting, surface crusts may form, which leads to aesthetic damage to the painting.^8^ To understand the local conditions under which these metal arsenates can form, further investigations are needed. This will not only help to understand the degradation of arsenic sulfide pigments but also to gain a deeper insight into the mechanisms of other pigment degradation reactions in paintings and the role of the medium herein.

## Conclusions

For a long time, arsenolite was considered to be the main degradation product of orpiment. Although we have confirmed that this reaction is triggered by direct light exposure, we here emphasize the importance of a better understanding of the formation of As(V) species. Multiple case studies have already shown that As(V) species are often present throughout the multi-layer system of oil paintings. In our study, we have observed that As(V) species can form within a period of weeks, and without the presence of (artificial) light. In our studies, we do not observe a direct transformation from solid As_2_O_3_ to As(V) species (reaction step D). We conclude that As(V) species are formed via As(III)_(aq)_ species, and As_2_O_3(s)_ is not always part of the degradation from orpiment to As(V) species. The As(V)_(aq)_ species are able to migrate very easily through different types of mediums and form solid metal arsenates. Our results are consistent with earlier research showing the release of arsenite and arsenate species from orpiment, without the formation of arsenolite. Looking back at Figure 2, this means that the transformation of orpiment in the presence of a medium either follows reaction steps B, E, and F (dissolution and oxidation followed by precipitation) or A, C, E, and F (formation of arsenolite, dissolution, and oxidation, followed by precipitation). The former can take place in either dark or light conditions, and the latter can only take place in the presence of light. In the absence of a medium but the presence of light, only reaction step A (orpiment to arsenolite) takes place.

A previous hypothesis was focused on the role of water in the migration of these species. In our study, we have not seen a significant difference in the migration of As(V) species under different RH conditions. Water-driven migration may still be a possibility, but our results show that the presence of an almost negligible amount of water would already be enough for the migration to take place. The short timeframe of the formation of As(V) species and the migration comes with concerns for the safety of paintings containing arsenic sulfide pigments. Contact with solvents and water facilitates the migration of As(V) species and thereby initiates the formation of new insoluble As-species. Conservators should be aware that there is a high probability of As(V) species being present in the varnish and other paint layers of arsenic–sulfide-rich areas of paintings.

## Supporting Information Available

Experimental procedures, materials and methods, SEM (-EDX) image, XANES, μ-XRF maps, MA-XRD pattern, and ATR-IR results (PDF)
