# Supplementary material for: Two Pathways for the Degradation of Orpiment Pigment (As2S3) Found in Paintings
Source: J Am Chem Soc. 2023 Apr 14;145(16):8847–59. doi: 10.1021/jacs.2c12271 (PMC10141271; doi:10.1021/jacs.2c12271)
Supplement: Supplementary file 1 — ja2c12271_si_001.pdf [file ja2c12271_si_001.pdf]

Supporting Information

Two Pathways for the Degradation of Orpiment Pigment (As_2S_3) Found in Paintings

Fréderique Broers[a,b,c,d*], Koen Janssens[c], Johanna Nelson Weker[e], Samuel M. Webb[e], Apurva Mehta[e], Florian Meirer[d] and Katrien Keune[a,b]

F.T.H. Broers, Dr. K. Keune

[a] Conservation & Science, Scientific Research, Rijksmuseum, Hobbemastraat 22, 1071 ZC Amsterdam, The Netherlands.

[b] Van't Hoff Institute for Molecular Sciences, University of Amsterdam, Science Park 904, 1090 GD Amsterdam, The Netherlands

[c] AXIS Antwerp X-ray Imaging and Spectroscopy laboratory, University of Antwerp, Groenenborgerlaan 171, 2020 Antwerp, Belgium

[d] Inorganic Chemistry & Catalysis, Debye Institute for Nanomaterials Science & Institute for Sustainable and Circular Chemistry, Utrecht University, Universiteitsweg 99, 3584 CG Utrecht, The Netherlands

[e] Stanford Synchrotron Radiation Lightsource, SLAC National Accelerator Laboratory, 2575 Sand Hill Rd, Menlo Park, CA, USA

Abstract: Paintings are complex objects containing many different chemical compounds that can react over time. The degradation of arsenic sulfide pigments causes optical changes in paintings. The main degradation product was thought to be white arsenolite (As2O3), but previous research also showed the abundant presence of As(V) species. In this study we investigate the influence of the presence of a medium on the degradation mechanism of orpiment (As2S3) using Synchrotron Radiation (SR)-based tomographic transmission X-ray microscopy (TXM), SR-based micro–X-ray Fluorescence (SR µ-XRF) and XANES spectroscopy. Upon direct illumination of dry orpiment powder using UV-visible light, only the formation of As2O3 was observed. When As2S3 was surrounded by a medium and illuminated, As2O3 was only observed in the area exposed to light while As(V) degradation species were found elsewhere in the medium. Without accelerated artificial light ageing, As(V)(aq) species are formed and migrate throughout the medium within weeks after preparation. In both scenarios, the As(V) species form via intermediate As(III)(aq) species and the presence of a medium is necessary. As(V)(aq) species can react with available cations to form insoluble metal arsenates, which induces stress within the paint layers (leading to e.g., cracks and delamination) or can lead to a visual change of the image of the painting.

Table of Contents

Experimental Procedures 2
Results and Discussion 4
References 9

Experimental Procedures

Overview samples

Table 1 Different conditions of the experiments performed with pure orpiment

Sample	Relative Humidity	Light ageing	Atmosphere
As2S3_Pure_LA_RH11	11	Yes	Air
As2S3_Pure_Dark_RH11	11	No	Air
As2S3_Pure_LA_RH90	95	Yes	Air
As2S3_Pure_Dark_RH90	95	No	Air
As2S3_Pure_LA_N2	-	Yes	N_2
As2S3_Pure_Dark_N2	-	No	N_2

Table 2 Overview of the samples made with $NaAsO_2$, orpiment and arsenolite and the different conditions

Sample	Relative Humidity	Light ageing	Atmosphere
$NaAsO_2$_dammar_dark	95	No	Air
As2S3_Egg_LA_RH11	11	Yes	Air
As2S3_Egg_LA_RH90	95	Yes	Air
As2S3_Egg_Dark	95	No	Air
As2O3_Egg_Dark	95	No	Air
$NaAsO_2$_Egg_Dark	95	No	Air
As2S3_Paraloid_LA_RH11	11	Yes	Air
As2S3_Paraloid_LA_RH90	93	Yes	Air
As2S3_Leadwhite_LA_45	45	Yes	Air

Pure pigment

For the samples consisting of pure pigment, some orpiment was placed in a small glass flask. These glass flasks were put into boxes with a saturated salt solution to maintain a certain relative humidity. Two different salt solutions were made: for a RH of 11% a saturated KOH solution, and for a RH of 95% a saturated salt solution of K_2SO_4. The models were artificially aged in an Opsytec Dr. Gröbel BS-02 irradiation chamber. The samples were aged for about 16 days under a mixed set of UV-A and cool day light lamps and 4 days of UV-B light lamps. Within this time the samples were exposed to an average of:

UV-A + cool day light lamps, per day: 130 mW/cm^2 (UVA sensor) 605 lxs (VIS sensor)

UV-B lamps, per day: 170 mW/cm^2 (UVA sensor) 640 mW /cm^2 (UVB sensor)

iButtons were used to monitor the RH and the temperature during the ageing. In the low RH boxes, the temperature fluctuated between 26 and 27 degrees Celsius during irradiation and the RH fluctuated between 14 and 18 %. In the high RH boxes, the temperature fluctuated between 27 and 29 degrees Celsius during irradiation and the RH fluctuated between 95 and 98 %.

Orpiment in medium

A few particles of orpiment were divided over a glass slide covered with PTFE tape. This tape was used for its inert and hydrophobic characteristics. A few drops of water diluted egg yolk (1:1) were applied on top of the orpiment particles to cover the particles.

The samples that were kept in the dark were kept at a high RH (95-98 %) for two weeks. Micro fragments were then taken from the samples and embedded in Technovit LC2000 (Heraeus Kulzer GmbH).

The models that were artificially light aged, were aged in an Opsytec Dr. Gröbel BS-02 irradiation chamber. The samples were aged for about 16 days under a mixed set of UV-A and cool day light lamps and 4 days of UV-B light lamps. Micro fragments were then taken from the samples and embedded in Technovit LC2000 (Heraeus Kulzer GmbH).

During the light ageing the samples were exposed to an average of:

UV-A + cool day light lamps, per day: 130 mW/cm^2 (UVA) 605 lxs (VIS)

UV-B lamps, per day: 170 mW/cm^2 (UVA) 640 mW /cm^2 (UVB)

iButtons were used to monitor the RH and the temperature during the ageing. In the low RH boxes, the temperature fluctuated between 26 and 27 degrees Celsius during irradiation and the RH fluctuated between 14 and 18 %. In the high RH boxes, the temperature fluctuated between 27 and 29 degrees Celsius during irradiation and the RH fluctuated between 95 and 98 %.

Paraloid B-72 was used as an alternative medium. Paraloid B-72 is an ethyl methyl acrylate co-polymer that is often used in conservation practices due to its stability and hardness. This medium was also applied with a few drops on top of the orpiment. The lighting and RH conditions were the same as described above.

Orpiment and lead white paint reconstructions

The paint reconstruction was prepared by Arie Wallert and Katrien Keune in 2016. The orpiment paint layer consists of a mixture of orpiment from Natural Pigments Rublev and orpiment synthesized by A.W. by the dry sublimation process. As binder, raw linseed oil (Kremer) was used. To enhance the drying properties of the paint, a small amount of alkyd oil (Talens) and glass beads provided by Erma Hermens

(Rijksmuseum Amsterdam) were added. The lead white paint was provided by Maartje Stols-Witlox and Emilie Froment (University of Amsterdam).
The paint layers were applied on canvas. The paint reconstructions were aged for 27 days using a XENON light ageing chamber. The XENON lamp had a strength of 105 lx and included a 320 nm cut-off filter. The relative humidity in the chamber was 40 % and the temperature 50° Celsius. The paint reconstruction was then kept for 10 months at a relative humidity of 43 %. A cross section was taken of the reconstruction and embedded in Technovit LC2000 (Heraeus Kulzer GmbH).

Preparation of cross-section of De Heem
Arie Wallert took a microsample of *Still life with Flowers in a Glass Vase* with a scalpel. The paint fragment was embedded in Technovit LC2000 (Heraeus Kulzer GmbH). For the SR analysis, the paint sample was decreased in dimension by FIB-SEM. This was performed by Martin Veselý at Utrecht University.

$NaAsO_2$_dammar_dark
Two drops of As(III) solution were applied on glass microscope slides with a concave. The slides were placed on a heating plate of 100 degrees Celsius to evaporate the water. Subsequently, the recrystallized As(III) was covered by applying a few drops of dammar resin on top. The sample was dried for 24 hours and afterwards kept at a high relative humidity of about 95 % for 8 days.

Methods
SEM-EDX
Scanning electron microscopy (SEM) studies of the pure orpiment pigment were performed on a XL30 SFEG high vacuum electron microscope (FEI, Eindhoven, The Netherlands)). Backscattered electron images of the cross-sections were taken at a 20 kV accelerating voltage, a 5 mm eucentric working distance and a spot size of 3, which corresponds to a beam diameter of 2.2 nm with a current density of approximately 130 pA. The pigment particles were adhered on copper tape, so no coating was necessary.

XANES and μ-XRF maps

X-ray Absorption Near Edge Structure (XANES) spectroscopy was performed at beamline 2-3 at the Stanford Synchrotron Radiation Lightsource (SSRL), SLAC National Accelerator Laboratory. BL2-3 is a bending magnet side station dedicated to X-ray imaging and micro X-ray absorption spectroscopy operating at an energy range from 5 to 24 keV. A Kirkpatrick–Baez mirrors system is used to achieve micro-focus with a beam size of ~2 × 2 μm2. The microscope consists of a double crystal monochromator [Si (111)] used for energy selection and scanning of the X-ray energy. Beamline 2-3 is equipped with a Vortex silicon drift detector and ionization chambers. Sample positions are controlled with submicron accuracy by a Newport Micro sample positioner. The samples were placed with double sided adhesive tape (Scotch® tape) in the sample holder and measured under ambient conditions and room temperature. Prior to the analysis of the paint cross-sections, powdered reference samples (described below) were prepared for bulk XANES analysis by dusting the finely ground powder onto Scotch® tape. Cross-section samples were kept in a vertical plane at 90° to the incident beam. The XANES consists of 130 energy points across the arsenic K-edge. Normalization of the XANES data was performed using Athena Software.[1] Multi energy μ-XRF maps of the samples were also gathered at BL 2-3 at SSRL. In total, 30 or 45 maps were taken across the As-K edge.

Data analysis of multi energy μ-XRF
To analyze the multi-energy μ-XRF maps, the XANES Wizard software was used.[2] To filter out pixels with a very low intensity of arsenic, and edge jump filter was applied to the data. The data was subsequently normalized.
PCA was carried out using the mean-centered data matrix (consisting of p pixels and E energies) and singular value decomposition (SVD) reducing the dimensionality of the data set from E to N dimensions. This reduction is achieved by using only the first N principal components (PCs), which explain most of the data's variance, but without losing significant information because higher PCs describe mainly the noise in the data set. Therefore, and also because the PCs are oriented to best describe the spread in the data, projecting the data to this N-dimensional principal component space highlights the pattern explained by the captured variance and effectively reduces noise. In this reduced space the distance between data points is a direct measure of the similarity of the XANES, i.e. the chemical phase, and can be used to cluster pixels according to their (Euclidean) distances from cluster centers (centroid linkage method, k-means clustering). This results in an effective grouping of pixels with similar XANES into k groups resulting in image segmentations. The number of clusters k has to be at least equal to N in order

to exploit all the information provided by the reduced PC space, but the data was intentionally over-clustered using to achieve a finer image segmentation in order to inspect various different stages of oxidation state transitions. The result of this clustering was used as an initial guess for a Gaussian Mixture Model (GMM). The GMM algorithm implements a data point density based clustering method using an expectation-maximization (EM) algorithm. Within the K-means clustering the assignment to a cluster is only based on the Euclidian distance to the centroid of the cluster. Within the GMM algorithm the point density in principal component space is incorporated. The result is that a certain data point can be attributed to different clusters with a certain weight, called a class-membership value. These weighted classifications are then used to reconstruct the average XANES for the different clusters. A more detailed description of the applied approach to process XANES image data can be found in references.[2–4]

Full-field transmission X-ray tomography

Full-field TXM measurements were performed at the wiggler beamline 6-2 at SSRL. The setup consists of a LN_2 cooled, fixed-exit double-crystal monochromator equipped with Si(111) to tune the beam energy and the X-ray microscope optimized for photon energies in the range of 5 to 14 keV with spatial and energy resolutions of 30 nm and $\Delta E/E = \sim 10^{-5}$ (Si(111) crystal), respectively. The microscope achieves a single flat FOV of about 15 x 15 μm^2 or 30 x 30 μm^2 (dependent on the optics used).
A tomography scan using a range from -90° to 89° with an angular increment of 1° was performed at BL 6-2c. After calibration of the energy on a copper foil, tomography was performed at 4 energies across the arsenic K-edge, namely at 11855 eV (pre-edge), 11871 eV (white line of As_2S_3), 11875 eV (white line of As(V)) and 12000 eV (post-edge). The selection of the energies was done before it was clear that the sample did not contain significant amounts of As^{5+} species. However, by comparing the ratio of the intensity at 11871 and 11875 eV, we can distinguish As_xS_y species from As_2O_3.
Due to the limited field of view (FOV) of 30 x 30 μm^2 in the full field method, 9 FOVs were collected and put together to image a large enough field of view ('mosaic imaging mode'). All data were processed using the TXM Wizard software.[2]

Data analysis of TXM data
The TXM Wizard software was used for processing TXM data. The reference images taken before and after tomography were averaged. These averaged reference files are used for a reference correction of the projection images. As the data were collected using mosaic mode, mosaic stitching was performed (using a phase correlation algorithm) to align overlapping areas from consecutive single-shot images in the mosaic. The projection images were manually aligned to a common feature observed in all projection images. An iterative algebraic reconstruction technique (i-ART) algorithm (i=20) was implemented to retrieve a 3D representation of the sample. The Avizo™ software was used to visualize the 3D reconstruction.

Results and Discussion

Before light ageing the orpiment, Scanning Electron Microscopy was performed on pure pigment adhered on Cu tape, shown in Figure S1a. These images show the expected structure of orpiment consisting of layers of sheets.[26,27] Figure S1b shows the same area as shown in Figure S1a after light ageing for two weeks under UV-B light (total exposure 10.35 kJ/cm^2, see SI). It clearly shows that the orpiment had shrunk in size and that new crystal structures formed. The octahedral crystals are arsenolite: energy-dispersive X-ray spectroscopy (EDX) analysis showed the presence of As and O and the absence of S in these crystals (See Fig. S2), and the same type of crystals were previously identified as arsenolite crystals by Meirer et al.[28] Figure S1d shows an area of the light-aged orpiment where many of such octahedral arsenolite crystals formed. A control sample was kept in the dark for two weeks and did not show any changes in structure after ageing (see Figure S3a,b for the SEM images taken from the same area before and after ageing).

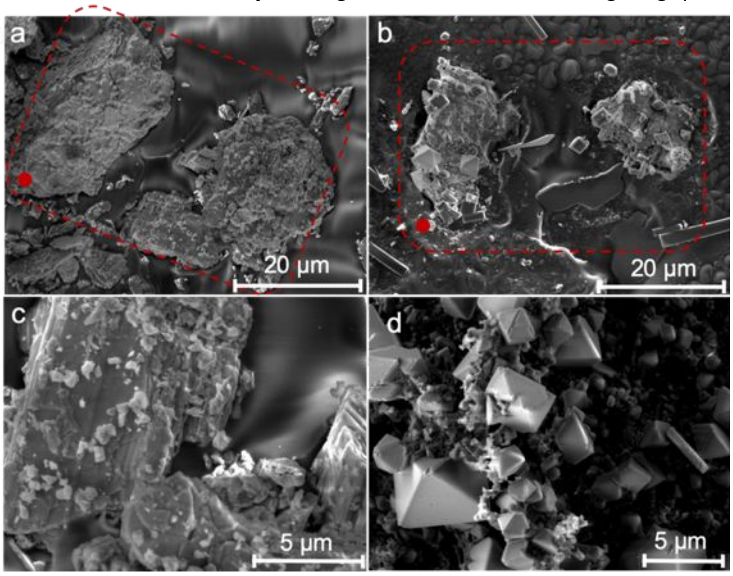

Figure S1. SEM Images showing a) an area of the orpiment on copper tape before ageing; b) the same area after light ageing, the red dashed area and dot are shown to guide the viewer in recognizing the area; c) area of orpiment with typical layered structure; and d) an area with many octahedral arsenolite crystals formed upon light ageing. The area shows a variety in arsenolite crystal size and textured material in between the crystals.

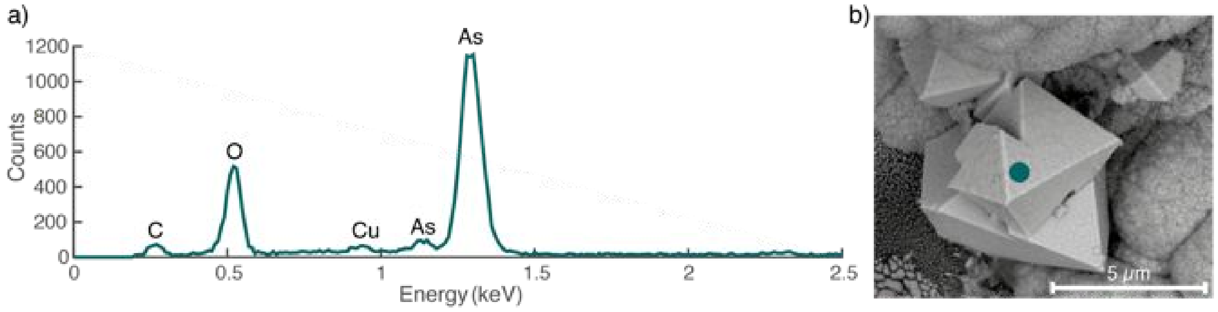

Figure S2. SEM-EDX measurement performed on arsenolite crystal. a) Result of the EDX measurement, showing the presence of mainly As and O in the measurement location. The copper is originating from the copper tape on which the pigment was placed. B) SEM image of the arsenolite crystal and the EDX location.

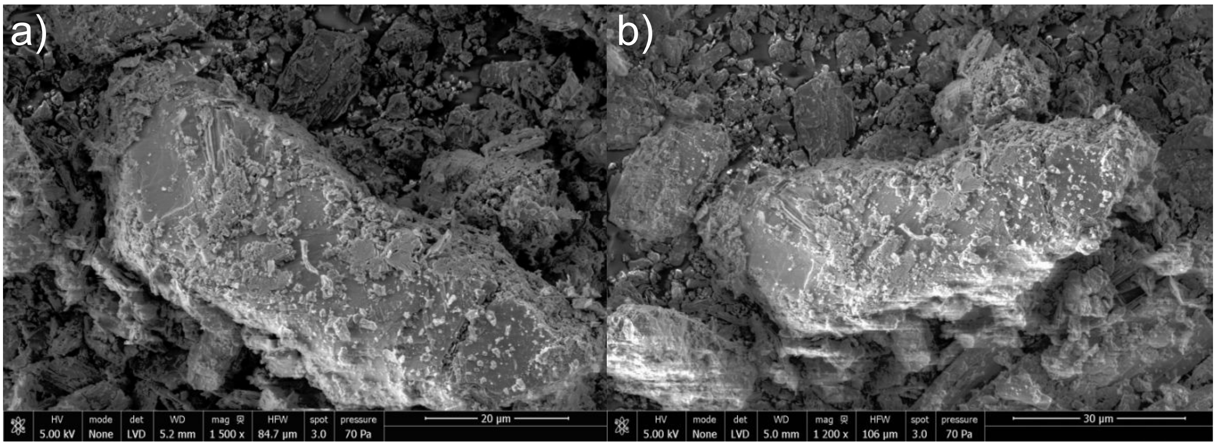

Figure S3. SEM images of orpiment on copper tape that was not exposed to artificial light ageing. a) SEM image at the start of the experiment b) SEM image of the sample after two weeks. No changes took place. (Note the difference in magnification).

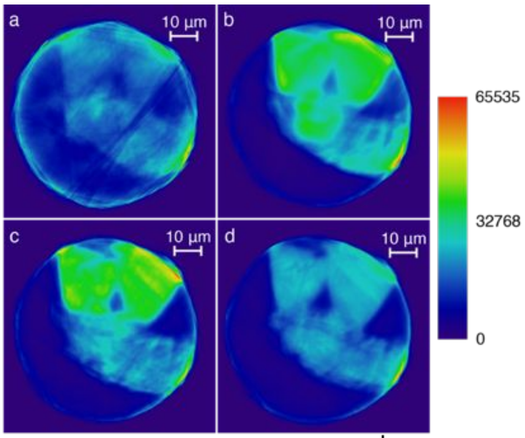

Figure S4. a) Virtual cross-section through the tomographic TXM at a) 11855 eV, b) 11871 eV, c) 11875 eV, and d) 12000 eV.

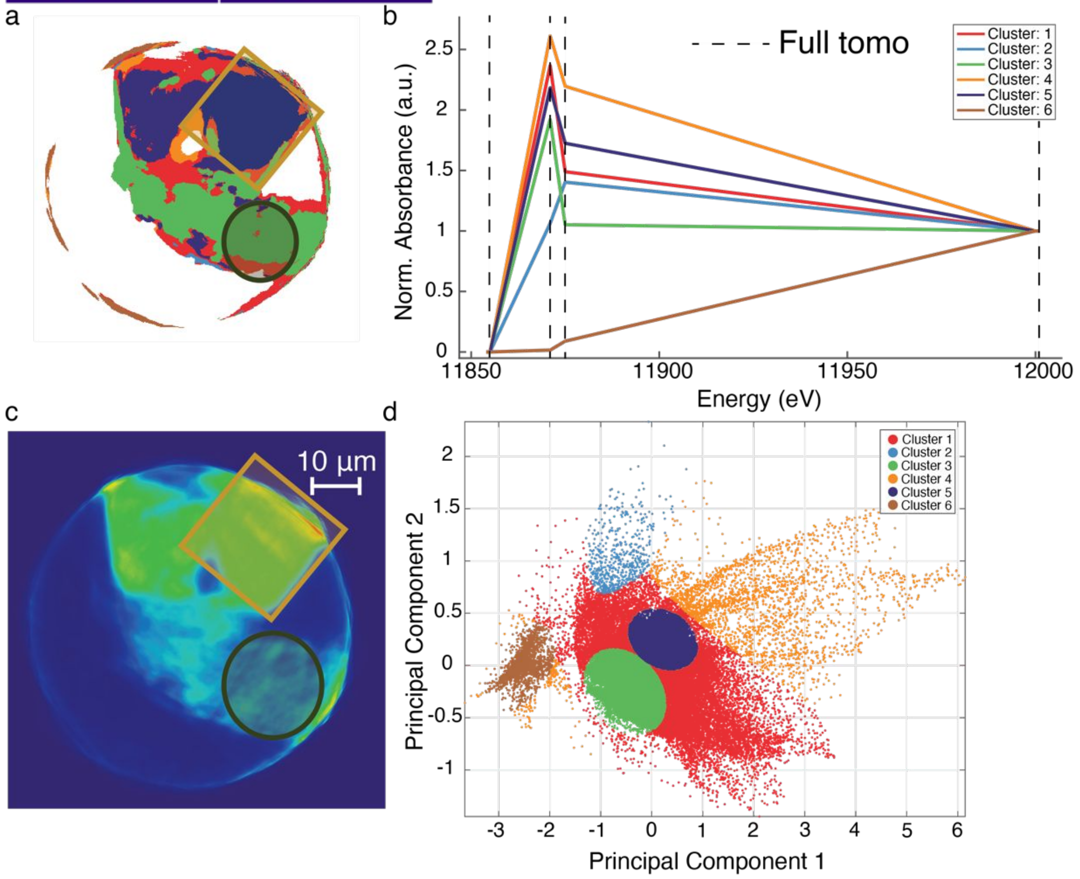

Figure S5. Results of analysis of the TXM data recorded at four energies: a) clustering result using 6 clusters (see SI for details) for segmenting the virtual cross-section displayed in figure 5; the square refers to the arsenolite area and the circle to the arsenic sulfide area identified by visual inspection of the 3D sample morphology (figure 4). b) 4-energy reconstructed XANES of the six clusters (dashed vertical lines indicate the energies employed), c) intensity map of the virtual cross-section at 11875 eV, and d) scatter plot of the two-dimensional principle component space used for clustering.

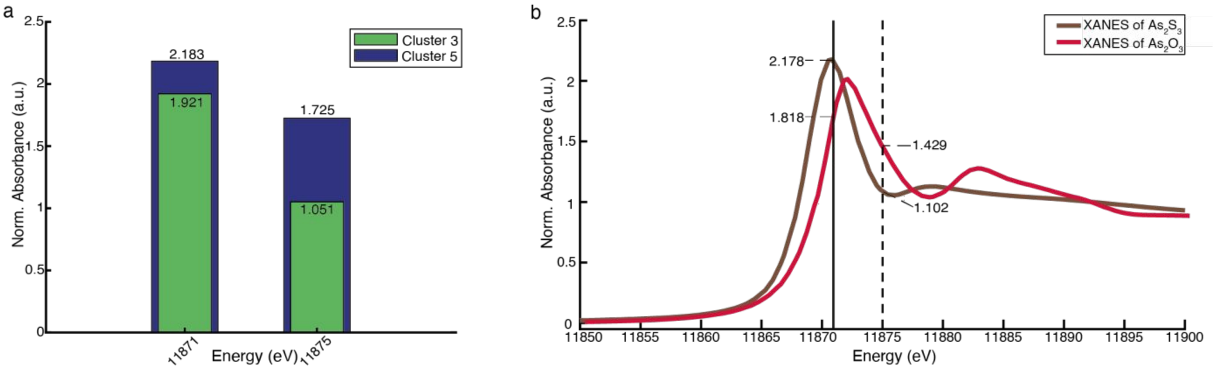

Figure S6. a) Bar plots of the normalized absorbance at 11871 and 11875 eV of the green and purple cluster in Fig. S6b. b) Reference XANES of orpiment (brown) and arsenolite (red). Reference spectra were also collected at beam line 2-3 at SSRL.

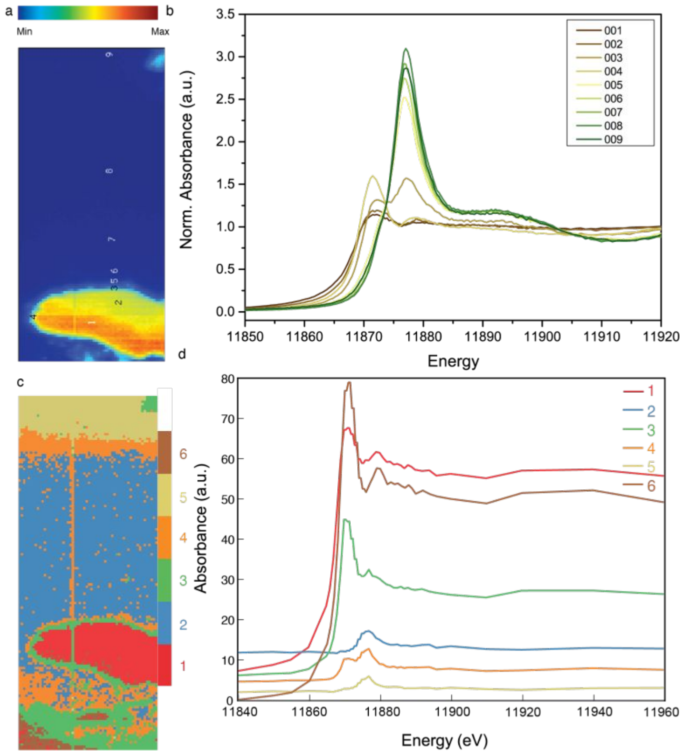

Figure S7. a) XRF map at 12 keV of cross section taken from the orpiment in egg sample kept at high RH, 1-9 indicate the spots where full XANES have been taken. Light ageing took place from the top. b) The normalized XANES 1-9. Spectrum 3 shows a shift in energy expected for As_2O_3 c) Multi-energy XRF maps were taken and this shows the clustered image that is the result of a PCA (using the first two PCs) followed by K-means clustering using six clusters and subsequent refinement by GMM d) reconstructed XANES of the six clusters. No normalization was performed for plotting as this gave a very poor result for the clusters with a weak As signal.

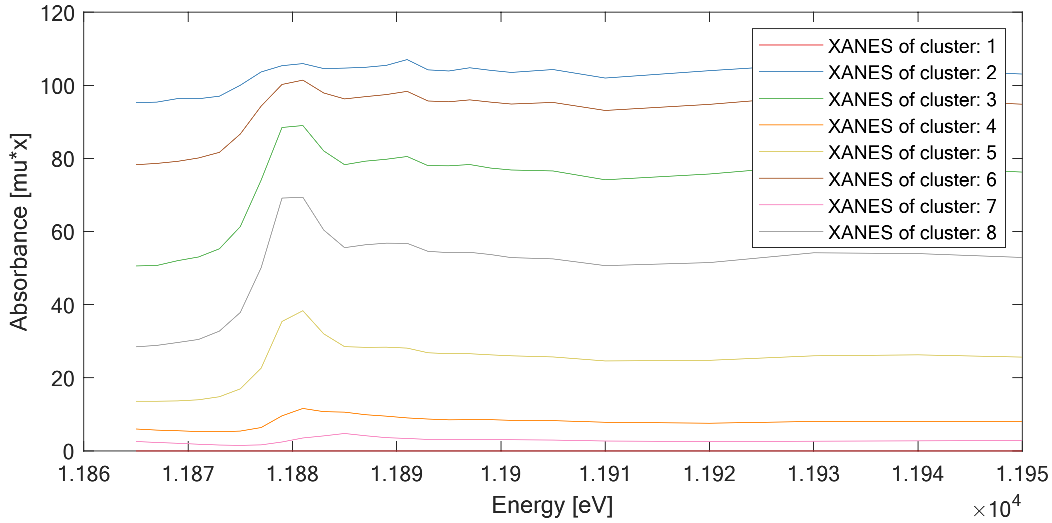

Figure S8. Non normalized reconstructed XANES of the 8 clusters of As_2S_3 in egg tempera.

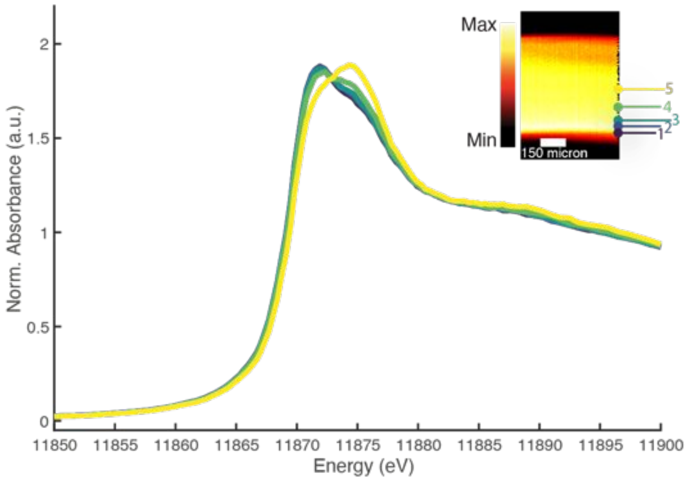

Figure S9. The inset shows the XRF map at 12 keV of As(III) salt (sodium(meta)arsenite, $NaAsO_2$) in a layer of egg tempera. 1-5 indicate the spots where full XANES spectra have been taken. The normalized XANES spectra 1-5 are shown, indicating the presence of As(III) and As(V) species. Spot 1 is where the arsenic salt was deposited on the PTFE tape.

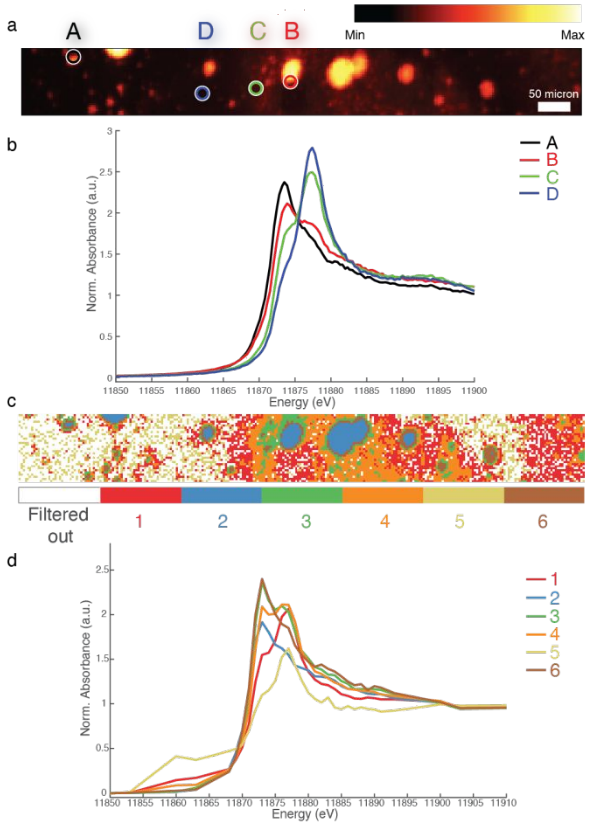

Figure S10. a) XRF map at 12 keV of $NaAs(III)O_2$ in a thin layer of dammar. A-D indicate the spots where full XANES spectra have been taken. b) The normalized XANES spectra A-D. c) Reconstructed and normalized XANES spectra of the six clusters. d) Clustering image that is the result of a PCA, K-means clustering and GMM.

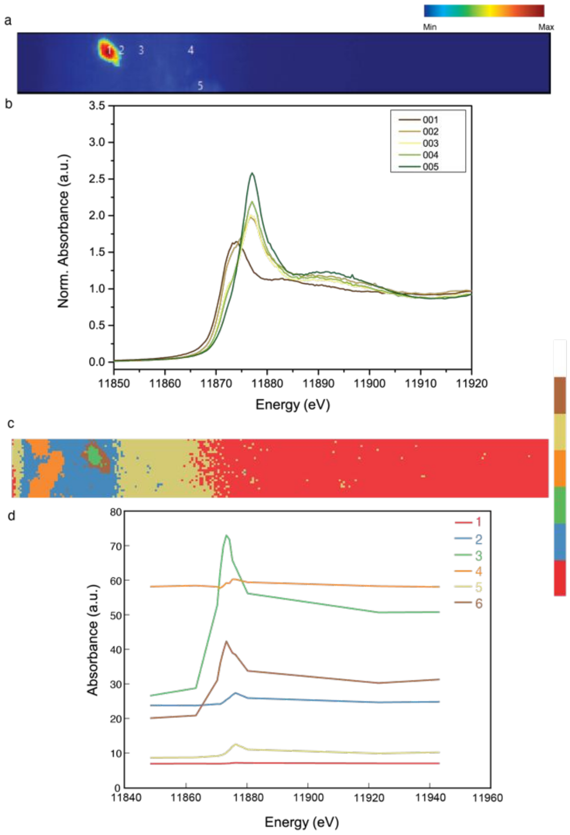

Figure S11. a) XRF map at 12 keV of cross section taken from the orpiment in Paraloid B-72 kept at low RH, 1-5 indicate the spots where full XANES have been taken. Light ageing took place from the right. b) The normalized XANES 1-5 c) Multi-energy XRF maps were taken and this shows the clustered image that is the result of a PCA (using the first three PCs) followed by K-means clustering using six clusters and subsequent refinement by GMM d) reconstructed XANES of the six clusters. No normalization was performed for plotting as this gave a very poor result for the clusters with a weak As signal.

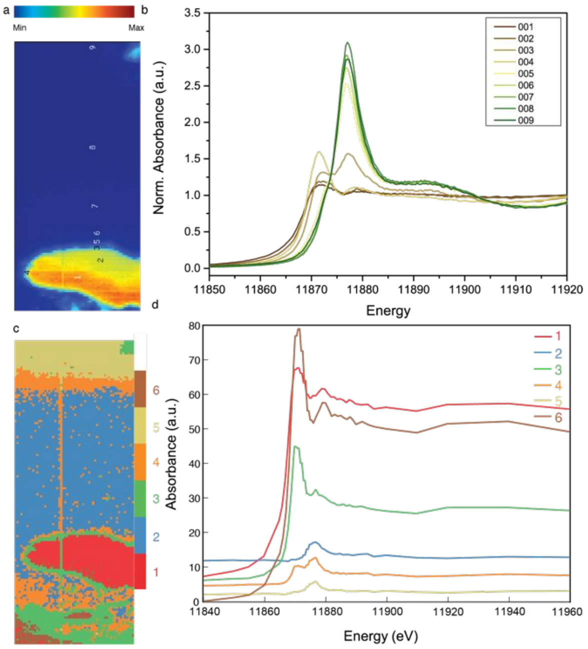

Figure S12. a) XRF map at 12 keV of cross section taken from the orpiment in Paraloid B-72 kept at high RH, 1-5 indicate the spots where full XANES have been taken. Light ageing took place from the left. b) The normalized XANES 1-5 c) Multi-energy XRF measurements were performed in a different area than the XANES spectra were taken. In this view, the light ageing was done from the right. This shows the clustered image that is the result of a PCA (using the first two PCs) followed by K-means clustering using six clusters and subsequent refinement by GMM d) reconstructed XANES of the six clusters. No normalization was performed for plotting as this gave a very poor result for the clusters with a weak As signal.

ATR-IR microscopy data of the paint reconstruction of orpiment on lead white

The cross-section of the paint reconstruction was analyzed with a Perkin Elmer Spotlight 400 FT-IT imaging system with a mercury cadmium telluride (MCT) detector. The spectral resolution of the spectral maps was 8 cm^{-1} and the pixel resolution was 1.56 μm. The top layer area shows the presence of the characteristic As-O peak at 797 cm^{-1}. The bottom layer shows the presence of the C-O vibration of lead white which is identified by the peak at 1378 cm^{-1}.

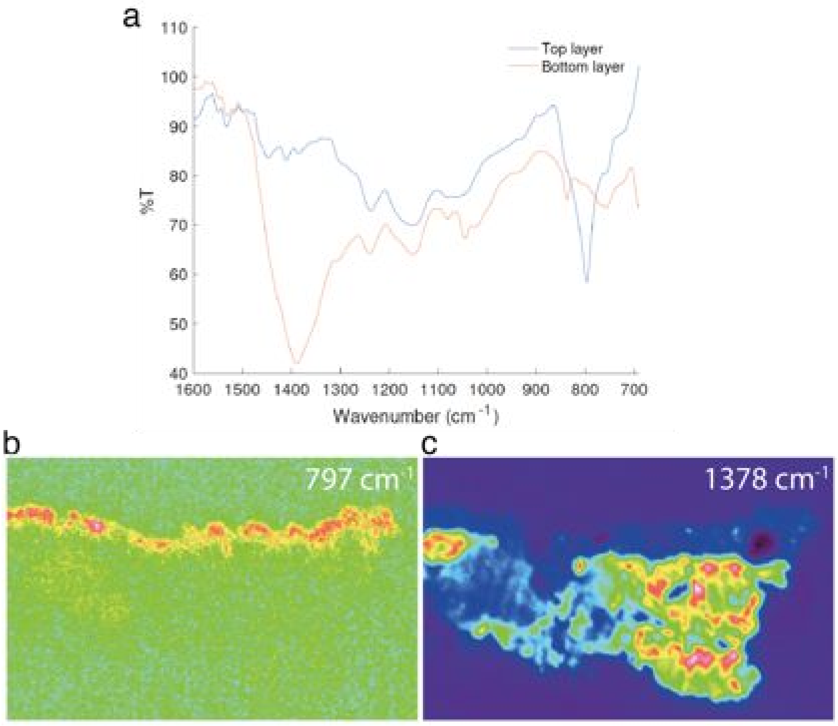

Figure S13. ATR-IR study of the paint reconstruction with a) FT-IR spectrum of the top layer (blue) and the bottom layer (red) b) FT-IR map of the peak intensity at 797 cm^{-1} (corresponding to As-O species) c) FT-IR map of the peak intensity at 1378 cm^{-1} (corresponding to C-O vibration of lead white)

MA-XRPD

A custom built laboratory MA-XRPD scanner with a monochromatic Cu-Kα (8.04 keV) X-ray source was used to analyze the sample. The X-ray source (IµS-CuHB, Incoatec GmbH, DE) was placed at an angle of 10 degrees and a distance of 20 cm relative to the sample in reflection geometry. A 2D diffraction detector (PILATUS 200K, DECTRIS Ltd., CH) was oriented at an angle of 45° with the sample surface.[5,6]

The MA-XRD results show the presence of orpiment, cerussite and hydrocerussite (original components of the lead white paint), and the presence of arsenolite. No crystalline As(V) species were identified.

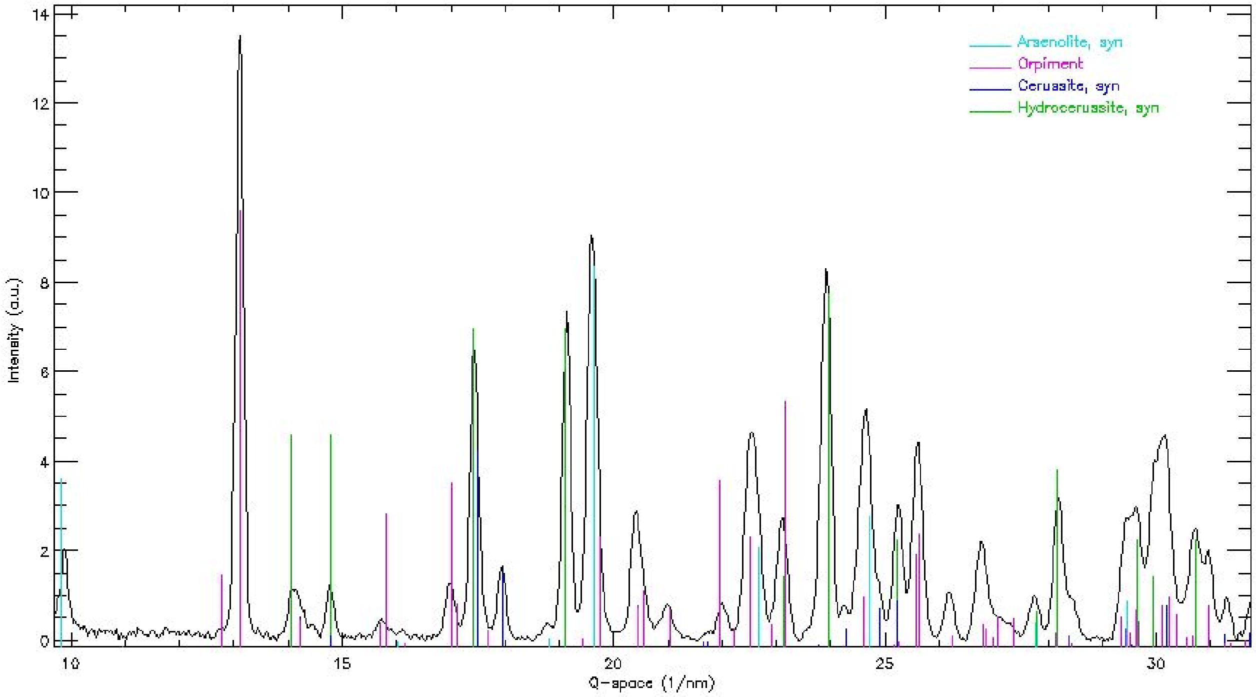

Figure S14. XRD Diffractogram of the paint reconstruction of an orpiment paint layer on a lead white ground layer.

References

(1) Ravel, B.; Newville, M. ATHENA, ARTEMIS, HEPHAESTUS: Data Analysis for X-Ray Absorption Spectroscopy Using IFEFFIT. *Journal of Synchrotron Radiation* **2005**, *12* (4), 537–541. https://doi.org/10.1107/S0909049505012719.

(2) Liu, Y.; Meirer, F.; Williams, P. A.; Wang, J.; Andrews, J. C.; Pianetta, P. TXM-Wizard: A Program for Advanced Data Collection and Evaluation in Full-Field Transmission X-Ray Microscopy. *Journal of Synchrotron Radiation* **2012**, *19* (2), 281–287. https://doi.org/10.1107/S0909049511049144.

(3) Meirer, F.; Liu, Y.; Pouyet, E.; Fayard, B.; Cotte, M.; Sanchez, C.; Andrews, J. C.; Mehta, A.; Sciau, P. Full-Field XANES Analysis of Roman Ceramics to Estimate Firing Conditions—A Novel Probe to Study Hierarchical Heterogeneous Materials. *Journal of Analytical Atomic Spectrometry* **2013**, *28* (12), 1870. https://doi.org/10.1039/c3ja50226k.

(4) Wise, A. M.; Weker, J. N.; Kalirai, S.; Farmand, M.; Shapiro, D. A.; Meirer, F.; Weckhuysen, B. M. Nanoscale Chemical Imaging of an Individual Catalyst Particle with Soft X-Ray Ptychography. *ACS Catalysis* **2016**, *6* (4), 2178–2181. https://doi.org/10.1021/acscatal.6b00221.

(5) De Meyer, S.; Vanmeert, F.; Vertongen, R.; van Loon, A.; Gonzalez, V.; van der Snickt, G.; Vandivere, A.; Janssens, K. Imaging Secondary Reaction Products at the Surface of Vermeer's Girl with the Pearl Earring by Means of Macroscopic X-Ray Powder Diffraction Scanning. *Heritage Science* **2019**, *7* (1), 1–11. https://doi.org/10.1186/s40494-019-0309-3.

(6) Vanmeert, F.; De Keyser, N.; Van Loon, A.; Klaassen, L.; Noble, P.; Janssens, K. Transmission and Reflection Mode Macroscopic X-Ray Powder Diffraction Imaging for the Noninvasive Visualization of Paint Degradation in Still Life Paintings by Jan Davidsz. de Heem. *Analytical Chemistry* **2019**, *91* (11), 7153–7161. https://doi.org/10.1021/acs.analchem.9b00328.
